# Transcriptomic Analysis Reveals That Excessive Thyroid Hormone Signaling Impairs Phototransduction and Mitochondrial Bioenergetics and Induces Cellular Stress in Mouse Cone Photoreceptors

**DOI:** 10.3390/ijms25137435

**Published:** 2024-07-06

**Authors:** Hongwei Ma, David Stanford, Willard M. Freeman, Xi-Qin Ding

**Affiliations:** 1 Department of Cell Biology, University of Oklahoma Health Sciences Center, 940 Stanton L. Young Blvd., BMSB 553, Oklahoma, OK 73104, USA; hongwei-ma@ouhsc.edu; 2 Genes & Human Disease Research Program, Oklahoma Medical Research Foundation, Oklahoma, OK 73104, USA; david-stanford@omrf.org (D.S.); bill-freeman@omrf.org (W.M.F.)

**Keywords:** thyroid hormone, retina, photoreceptor, cone, phototransduction, scRNAseq

## Abstract

Thyroid hormone (TH) plays an essential role in cell proliferation, differentiation, and metabolism. Experimental and clinical studies have shown a potential association between TH signaling and retinal degeneration. The suppression of TH signaling protects cone photoreceptors in mouse models of retinal degeneration, whereas excessive TH signaling induces cone degeneration, manifested as reduced light response and a loss of cones. This work investigates the genes/transcriptomic alterations that might be involved in TH-induced cone degeneration in mice using single-cell RNA sequencing (scRNAseq) analysis. One-month-old C57BL/6 mice received triiodothyronine (T3, 20 µg/mL in drinking water) for 4 weeks as a model of hyperthyroidism/excessive TH signaling. At the end of the experiments, retinal cells were dissociated, and cell viability was analyzed before being subjected to scRNAseq. The resulting data were analyzed using the Seurat package and visualized using the Loupe browser. Among 155,866 single cells, we identified 14 cell clusters, representing various retinal cell types, with rod and cone clusters comprising 76% and 4.1% of the total cell population, respectively. Cone cluster transcriptomes demonstrated the most alterations after the T3 treatment, with 450 differentially expressed genes (DEGs), accounting for 38.5% of the total DEGs. Statistically significant changes in the expression of genes in the cone cluster revealed that phototransduction and oxidative phosphorylation were impaired after the T3 treatment, along with mitochondrial dysfunction. A pathway analysis also showed the activation of the sensory neuronal/photoreceptor stress pathways after the T3 treatment. Specifically, the eukaryotic initiation factor-2 signaling pathway and the cAMP response element-binding protein signaling pathway were upregulated. Thus, excessive TH signaling substantially affects cones at the transcriptomic level. The findings from this work provide an insight into how excessive TH signaling induces cone degeneration.

## 1. Introduction

Rod and cone photoreceptors play a central role in vision. Rods are responsible for dim light vision, whereas cones are responsible for bright light, color vision, and visual acuity. Rods and cones degenerate in a variety of pathological conditions, including inherited retinal degenerative diseases such as retinitis pigmentosa, Leber congenital amaurosis (LCA), and cone–rod dystrophies and age-related retinal degeneration such as age-related macular degeneration (AMD) and diabetic retinopathy. Inherited retinal degenerative diseases affect approximately 1 in 3000 individuals worldwide, whereas AMD is the leading cause of blindness among the older population.

Thyroid hormone (TH) signaling regulates cell proliferation, differentiation, and metabolism [1,2,3]. TH has also been associated with cell death/survival. TH signaling is the main driving force in apoptotic tissue remodeling during anuran metamorphosis [4,5]. TH signaling is associated with the apoptosis of a variety of human cells, including lymphocytes [6], breast cancer cells [7], HeLa cells [8], and pituitary tumor cells [9]. Excessive TH signaling has been shown to induce auditory defects/cochlear degeneration [10] and cerebellum degeneration [11] in mice. TH signaling abnormality has also been linked to neurodegenerative conditions/dementia in elderly individuals [12,13,14], including human cone/retinal diseases. Clinical studies show that an elevated TH level in circulation is associated with an increased incidence of AMD [15,16,17,18,19,20,21,22,23,24]. In line with this, an optical coherence tomography evaluation has shown macular thinning in patients with thyroid-associated ophthalmopathy [25,26].

In the retina, TH signaling is well known for its regulation in cone opsin expression/patterning and cone development. It suppresses the expression of S-opsin (short-wave-sensitive opsin 1), induces the expression of M-opsin (medium-wave-sensitive opsin 1) [27,28,29], and controls the dorsal–ventral gradient expression of cone opsins [27]. TH signaling also regulates cone survival/viability. The suppression of TH signaling by antithyroid treatment [30]; the inhibition of Dio2 [31,32], the enzyme responsible for converting the prohormone thyroxine (T4) to the active hormone triiodothyronine (T3); the overexpression of Dio3 [32,33], the enzyme responsible for degrading T3; or the deletion of the TH receptor [34] reduces cone degeneration in mouse models of LCA and cone dystrophy/achromatopsia and a chemically induced mouse model of AMD [35,36]. In contrast, stimulating TH signaling by T3 treatment [30,37,38] or the deletion of Dio3 [38] induces cone death in mice, accompanied with cellular oxidative stress, necroptosis, and inflammation. Moreover, cone degenerating retinas show an increased expression of the TH receptor [35] and iodothyronine deiodinases [32], suggesting that TH signaling activity is locally elevated and may play a role in disease progression.

The present work investigated the genes/transcriptomic alterations that might be involved in TH-induced cone degeneration in mice using single-cell RNA sequencing (scRNAseq) and a subsequent ingenuity pathway analysis (IPA). We found that cone cluster transcriptomes were at the top after T3 treatment. The IPA revealed that treatment with T3 suppressed phototransduction and oxidative phosphorylation, accompanied with mitochondrial dysfunction, and induced cellular stress/death pathways. This work, using a mouse model of hyperthyroidism, provides an insight into how excessive TH signaling induces cone degeneration.

## 2. Results

### 2.1. scRNAseq of Retinas Prepared from Control and T3-Treated Mice

**Figure 1:**
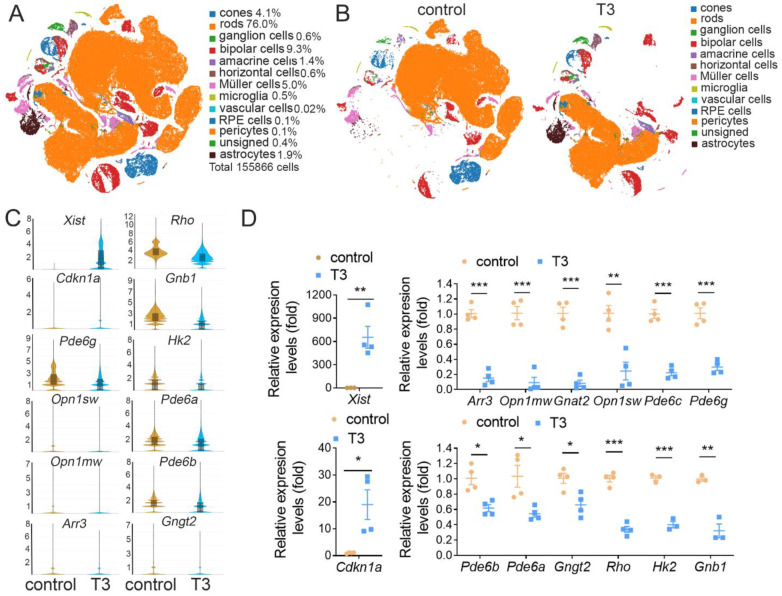
scRNAseq of mouse retinas after T3 treatment. (**A**) t-SNE plot of all libraries and percentage of each cell type in whole dataset. Cells are colored based on cell-type clusters. (**B**) t-SNE plot showing cell type distribution in retinas of control and T3-treated mice. (**C**) Violin plots from scRNAseq showing expression profiles of representative genes in retinas of control and T3-treated mice. (**D**) qRT-PCR data showing mRNA fold changes in representative genes in retinas of control and T3-treated mice. Data are presented as means  ±  SEM from 4 individual mice per group (* *p*  <  0.05, ** *p*  <  0.01, and *** *p* < 0.001 compared with their respective untreated controls).

To obtain transcriptomes of individual cells in the retina after the stimulation of TH signaling, droplet sequencing was conducted in the retinas of control mice and mice that were treated with T3 (20 µg/mL via drinking water) for 4 weeks. This treatment increases the serum T3 level by about 5–8-fold [37]. Retinas from four mice in each group were used. Retinal cells were dissociated/isolated using cell dissociation medium, and the average cell viability was about 57.5% according to flow cytometry. Following preprocessing and quality control, we identified 155,866 single cells that were grouped into 13 clusters with 918 median genes and 1465 median unique molecular identifiers per cell. The clusters included cells from both the control and T3-treated retinas. The clusters were annotated using a curated set of known marker genes (Table 1) representing different types of retinal cells. The cluster analysis showed that rods represent 76.0% of the total cell population, cones represent 4.1%, and bipolar cells, amacrine cells, Müller cells, and astrocytes represent 9.3%, 1.4%, 5.0%, and 1.9%, respectively (Figure 1A; Table 1). Treatment with T3 greatly altered the expressions of the genes in the retinal cells. The profound alteration in gene expression was shown on the t-SNE plots (Figure 1B). There were 144 differentially expressed genes (DEGs) and 2 unmapped genes in all retinal cells prepared from the mice treated with T3 compared with the untreated controls (Supplementary Table S1). Figure 1C shows the scRNAseq detection of the expressions of the representative retinal cell-specific genes, e.g., *Opn1sw*, *Opn1mw*, and *Arr3* for cones and *Rho*, *Pde6a*, *Pde6b*, *Gngt2*, and *Gnb1* for rods, and the universally expressed genes, e.g., *Xist* and *Cdkn1a*. We performed qRT-PCR to verify the scRNAseq DEGs. The qRT-PCR data were consistent with the scRNAseq data (Figure 1C,D). Thus, our scRNAseq identified 13 cell clusters in the retina with rods representing 76.0% of the total cell population, and cones, bipolar cells, amacrine cells, Müller cells, and astrocytes representing 4.1%, 9.3%, 1.4%, 5.0%, and 1.9%, respectively.

### 2.2. Cone Transcriptomes Are at the Top among Retinal Cells after T3 Treatment

**Figure 2:**
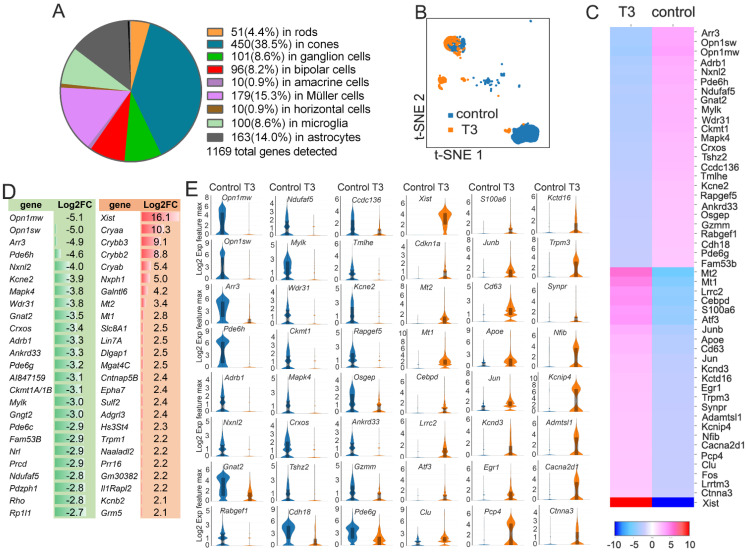
Cone transcriptomes are at the top among retinal cells after the T3 treatment. (**A**) A pie plot showing the number and percentage of DEGs in different retinal cell types after the T3 treatment. (**B**) A t-SNE plot showing the cone cell distribution profiles in the retinas of the control and T3-treated mice. (**C**) A heatmap showing the top 50 DEGs in cones after the T3 treatment. (**D**) A diagram showing the log2FC change amplitudes of the top 50 DEGs in cones after the T3 treatment. (**E**) Violin plots showing the change amplitudes of the top 48 DEGs in cones after the T3 treatment.

The extraction of DEGs in each cell cluster was conducted, and the data show that treatment with T3 greatly altered the expressions of the genes in the cones. There were 450 DEGs, representing 38.5% of the total amount of DEGs detected, in the cones after the T3 treatment based on the change fold and *p*-value of 0.05. The numbers of DEGs in the rods, bipolar cells, ganglion cells, Müller cells, microglia, and astrocytes were 51 (4.4%), 96 (8.2%), 101 (8.6%), 179 (15.3%), 100 (8.6%), and 163 (14%), respectively (Figure 2A; Table 1). Collectively, a total of 1169 genes were detected in 11 cell clusters (Table 1). The profound alteration of the gene expression in the cones after the T3 treatment is also shown on the t-SNE plots (Figure 2B). Thus, cone transcriptomes were at the top among retinal cells after the T3 treatment. Treatment with T3 led to the upregulation of 229 DEGs and the down-regulation of 221 DEGs in cones (Supplementary Table S2). The top 25 down-regulated DEGs included genes involved in phototransduction, such as *Arr3*, *Opn1sw*, *Opn1mw*, and *Pde6h*, and the top 25 upregulated DEGs included genes involved in metabolism and transcriptional regulation, such as *Mt2*, *Mt1*, *Lrrc2*, and *Cebpd*, and genes encoding the long non-coding RNAs, such as *Xist*, which mediates chromosome-wide gene silencing (Figure 2C–E).

### 2.3. Treatment with T3 Suppresses the Cone Phototransduction Pathway

**Figure 3:**
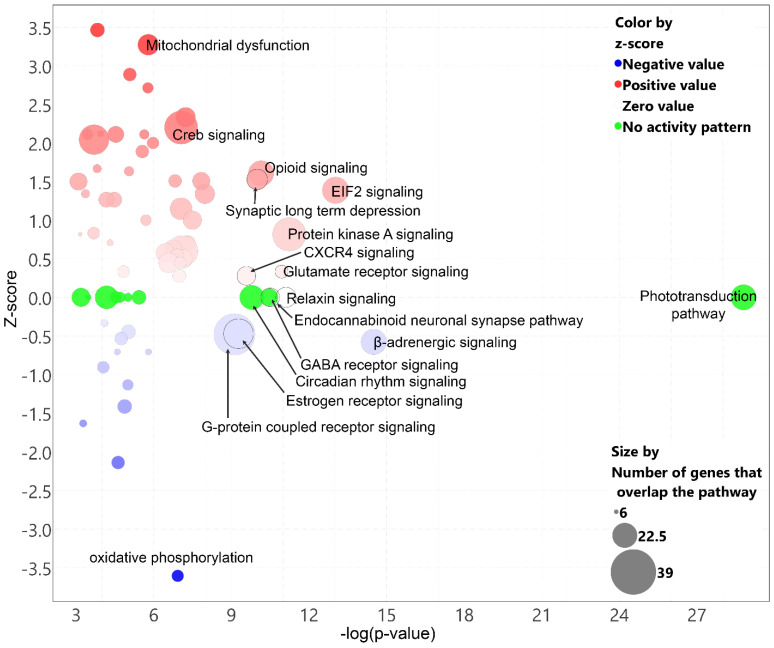
Treatment with T3 alters various canonical pathways in cones. Figure shows bubble chart of canonical pathways that were altered in cones after T3 treatment. Chart was generated based on *p*-value, z-score, and number of DEGs that overlap pathways.

**Figure 4:**
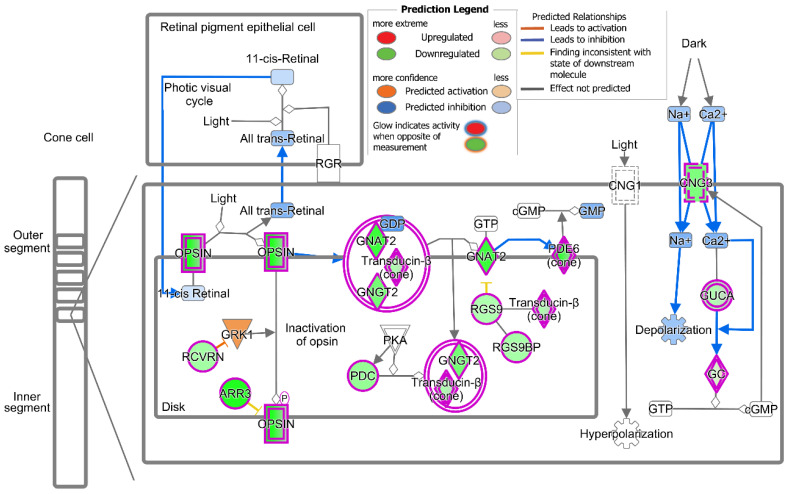
Treatment with T3 suppresses cone phototransduction. Figure shows IPA of phototransduction pathway in cones after T3 treatment, indicating down-regulation of numerous DEGs in cone phototransduction.

A pathway analysis of the DEGs (IPA) in cones after the T3 treatment revealed 17 altered pathways after the T3 treatment. The down-regulation of the phototransduction pathway was identified at the top of the list based on the log(*p*-value) ([−log(*p*-value) = 28.8, ratio = 0.463], Table 2, Figure 3), which was illustrated by the down-regulation of 24 DEGs, including *Opn1sw*, *Opn1mw*, *Arr3*, *Pde6h*, *Gnat2*, and *Cngb3* (Figure 4; Supplementary Table S3). These data suggest that treatment with T3 suppressed the cone phototransduction pathway.

### 2.4. Treatment with T3 Suppresses Oxidative Phosphorylation in Cones and Induces Mitochondrial Dysfunction

**Figure 5:**
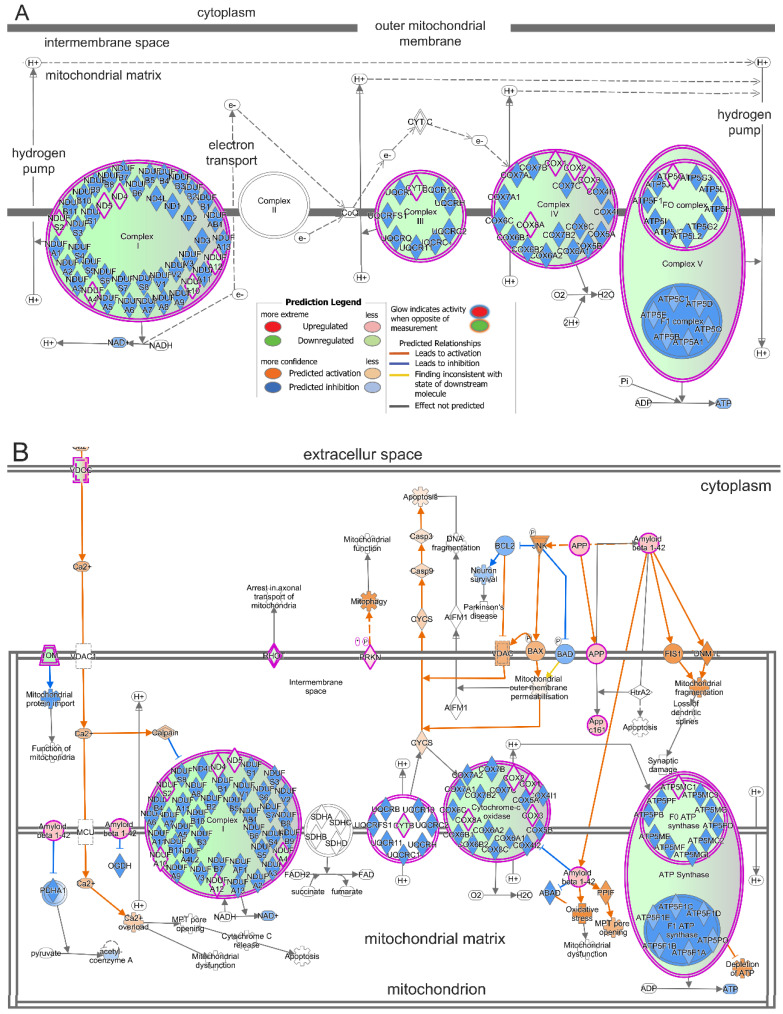
Treatment with T3 suppresses oxidative phosphorylation in cones and induces mitochondrial dysfunction. (**A**) IPA of oxidative phosphorylation pathway in cones after T3 treatment. DEGs were detected in 4 out of 5 oxidative phosphorylation complexes. (**B**) IPA of mitochondrial dysfunction pathway in cones after T3 treatment. There were 21 DEGs in pathway. Changes in these genes are likely involved in multiple aspects of mitochondrial function, including oxidative phosphorylation complexes, mitochondrial electron transportation, mitochondrial outer membrane permeabilization, mitochondrial fragmentation/fission, mitophagy, and cellular/mitochondrial calcium uptake.

Among the top down-regulated genes were also those involved in oxidative phosphorylation and mitochondrial function, e.g., *Atp5mc1*, *Ndufa12*, *Mt-atp6*, *Mt-co2*, *Mt-nd4*, and *Cox8a*. The IPA based on both the −log(*p*-value) and the z-score [−log(*p*-value) = 6.93, z-score = −3.61, and ratio = 0.117] revealed that oxidative phosphorylation was greatly suppressed (Table 2; Figure 3). There were 13 down-regulated DEGs, which are involved in four out of the five mitochondrial oxidative phosphorylation complexes. Proteins encoded by *Mtnd4*, *Mtnd5*, *Ndufa4*, *Ndufa10*, *Ndufa12*, and *Ndufs2* are located in complex I; MTCYB, encoded by Mtcyb, is located in complex III; proteins encoded by *Mtco1*, *Mtco2*, *Mtco3*, and *Cox8a* are located in complex IV; and ATP5G1, encoded by Atp5mc1, is located in complex V (Figure 5A; Supplementary Table S4). In line with the suppression of oxidative phosphorylation, mitochondrial dysfunction was detected as one of the top three significantly induced cellular events [−log(*p*-value) = 5.79, z-score = 3.27, and ratio = 0.061] (Table 2; Figure 3). There were 21 DEGs in the mitochondrial dysfunction pathway. Changes in these genes are likely associated with the inhibition of oxidative phosphorylation complexes (*Atp5mc1*, *Ndufa12*, *Mt-atp6*, *Mt-co2*, *Mt-nd4*, and *Cox8a*), mitochondrial electron transportation (*Rohot1*), and mitochondrial outer membrane permeabilization (*Tomm20*) (Figure 5B; Supplementary Table S5). The changes may also be related to the enhancement in mitochondrial fragmentation/fission (*App*), mitophagy (*Prkn*), and cellular/mitochondrial calcium uptake (*Cacna1d*, *Cacna1h*, and *Cacna2d1*) (Figure 5B; Supplementary Table S5). These results suggest that treatment with T3 suppressed oxidative phosphorylation in cones and induced mitochondrial dysfunction.

### 2.5. Treatment with T3 Induces Cellular Stress Pathways

**Figure 6:**
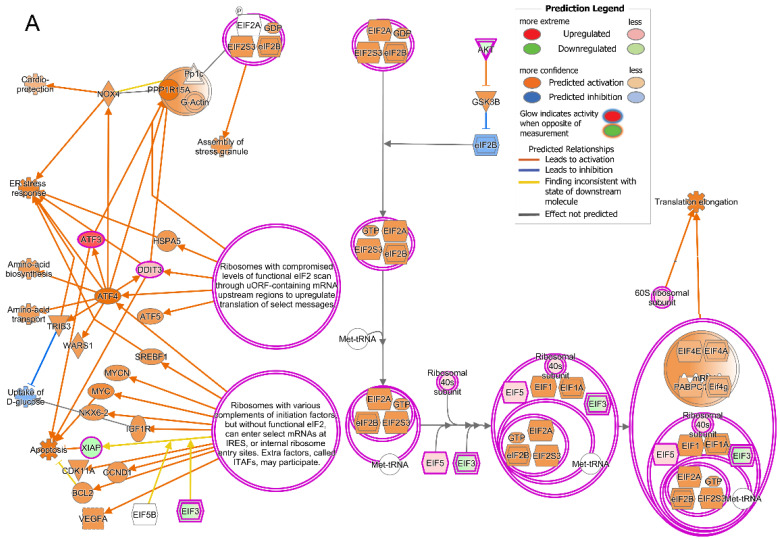

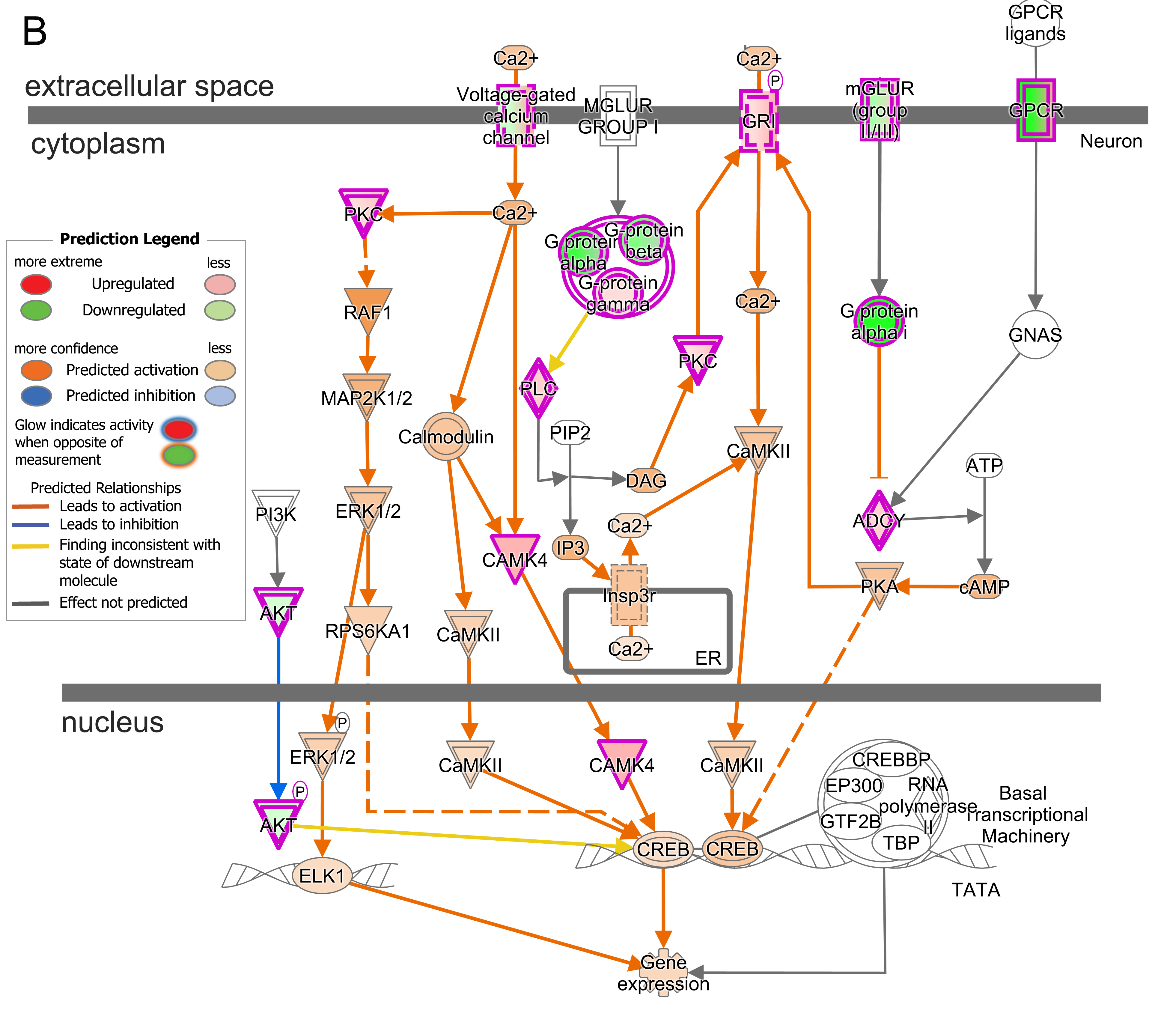
Treatment with T3 induces cellular stress responses in cones. (**A**) Treatment with T3 induces EIF2 pathway. Shown is IPA of EIF2 pathway in cones after T3 treatment. There were 26 DEGs in pathway. (**B**) Treatment with T3 induces CREB pathway. Shown IPA of CREB pathway in cones after T3 treatment. There were 16 DEGs in pathway.

The IPA also showed that two cellular stress pathways were activated in the cones after the T3 treatment. This includes eukaryotic initiation factor-2 (EIF2) signaling [−log(*p*-value) = 13.0, z-score = 1.39, ratio = 0.115] and cAMP response element-binding protein (CREB) signaling [−log(*p*-value) = 7.1, z-score = 2.2, ratio = 0.05] (Figure 3; Table 2). There were 26 DEGs in EIF2 signaling. Among these DEGs, 18 are known to encode L and S ribosomal proteins (Figure 6A; Supplementary Table S6). There were 32 DEGs in CREB signaling. Among these DEGs, *Lgr5* and *Adcy2* are known to activate CREB through PKA; *Plcb3* and *Plcl1* activate phospholipase C; *Prkca* and *Prkcb* activate PKC; *Gria1*, *Gria2*, *Grid1*, *Grik1*, *Grm7*, and *Grm8* may activate CaMKII; and *Cacna1d*, *Cacna1f*, *Cacna1h*, and *Cacna2d1* may activate *CAMK4* (Figure 6B; Supplementary Table S7). These data suggest that treatment with T3 induced cellular stress pathways in cones.

### 2.6. Prominent Pathway Networks and Upstream Regulators in Cones after T3 Treatment

The pathway network analysis yielded two networks. The first network was related to developmental disorders, hereditary disorders, and ophthalmic disease. Genes *Nr2e3*, *Nrl*, and *Crx*, involved in retinal development, were down-regulated after T3 treatment, implying developmental defect/disorder and hereditary disease (Supplementary Figure S1A). Also, genes involved in cone phototransduction, including *Pde6c*, *Pde6h*, and *Cngb3*, were down-regulated after T3 treatment, implying that there was a defect/disorder in cone phototransduction (Supplementary Figure S1A). The second network was involved in cell death and survival, cellular compromise, and neurological disease. The down-regulation of *Opn1sw*, *Opn1mw*, and *Arr3* was linked to decreased photoreceptor function and cell death (Supplementary Figure S1B). Genes involved in cellular compromise/stress, including *Dnajc5*, *Add1*, *Add3*, Rabgef1, *Grm8*, *Ndfip1*, *Laptm4b*, *Clathrin*, *Ccdc136*, *Ccdc88a*, and *Glmn*, were also down-regulated (Supplementary Figure S1B). In contrast, the *Stat3* and *Otx2* genes, which are involved in development, death signaling, and diseases, were upregulated (Supplementary Figure S1B). The gene network summary and pathway overlap were analyzed, showing high levels of interaction among the pathways (Supplementary Figure S2).

The upstream regulator analysis identified six regulators that were inhibited and one regulator that was activated in the cones after the T3 treatment. The inhibited upstream regulators included *Rabgef1* (log2FC = −2.28, *p* = 6.05 × 10^−16^), *Crx* (log2FC = −0.81, *p* = 5.07 × 10^−3^), *Icmt*, *Rax*, *Esrrb*, and *Gucy2f* (Supplementary Figure S3). These upstream modulators down-regulate the target genes, e.g., *Nrl*, *Rho*, *Cngb3*, *Prph2*, *Pde6b*, *Gnat1*, *Gucy2d*, *Pde6g*, *Aipl1*, and *Cdhr1*, which, in turn, decrease the function of retinal cells/photoreceptors, reduce the quantity of photoreceptors, and together with *Cx3cl1*, activate the death pathways (Supplementary Figure S3).

### 2.7. Transcriptomic Alterations in Rods after T3 Treatment

**Figure 7:**
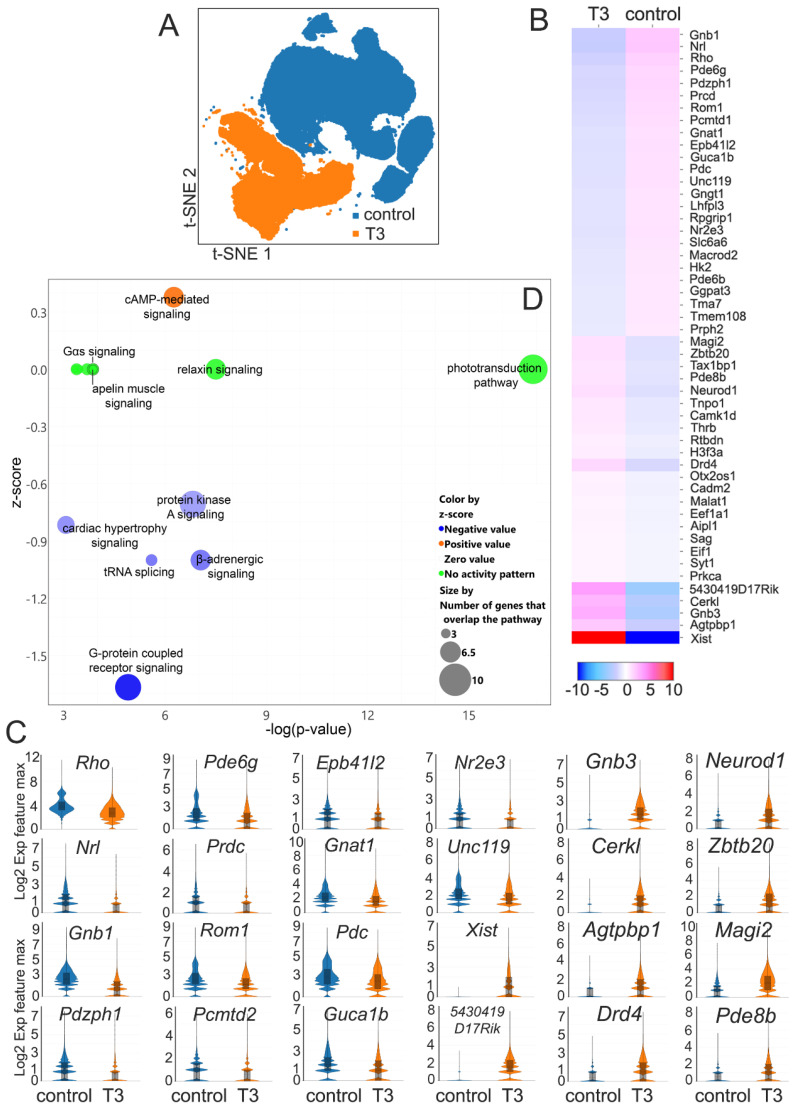
Transcriptomic alterations in rods after T3 treatment. (**A**) A t-SNE plot showing rod cell distribution profiles in the retinas of the control and T3-treated mice. (**B**) A heatmap showing DEGs in rods after the T3 treatment. (**C**) Violin plots showing the change amplitudes of the top 24 DEGs in rods after T3 treatment. (**D**) The IPA of the canonical pathways in rods after the T3 treatment. Shown is a bubble chart of the canonical pathways that were altered after the T3 treatment. This chart was generated based on the *p*-value, z-score, and the number of DEGs that overlap the pathways.

A transcriptomic analysis was conducted with rod clusters, showing 51 DEGs after the T3 treatment (Figure 2A; Supplementary Table S8). The alterations in gene expression in rods after the T3 treatment was also shown on the t-SNE plots (Figure 7A). Among the DEGs, there were 17 up- and 34 down-regulated genes after the T3 treatment. Many of these genes, e.g., *Rho*, *Gnb1*, *Gnb3*, *Pde6a*, *Pde6b*, *Pde6g*, *Pdc*, *Gnat1*, and *Guca1b*, are involved in phototransduction (Figure 7B,C). Genes involved in retinal development and differentiation, e.g., *Nrl*, *Nr2e3*, *Zbtb20*, and *Neurod*; in the morphogenesis of photoreceptor outer segment/disk, e.g., *Rom1*; and in the synaptic neurotransmitter release, e.g., *Unc119*, were also identified (Figure 7B,C). The results suggest that phototransduction was the top down-regulated pathway and cAMP-mediated signaling was the top upregulated pathway after T3 treatment (Figure 7D).

## 3. Discussion

The present work examined the genes/transcriptomic alterations that might be involved in TH-induced cone degeneration in mice using scRNAseq and a pathway/network analysis. Our findings reveal that excessive TH signaling has an adverse impact on cone phototransduction, mitochondrial bioenergetics, and cellular homeostasis/viability.

Our scRNAseq identified 13 cell clusters in the retina, which were, in turn, classified by cell type using a curated set of known cell identity marker genes. The cluster analysis data are highly consistent with previous reports using immunochemical/morphological approaches [39] and nanoliter droplets [40] (see Supplementary Table S9).

The cone transcriptomes demonstrated the most striking changes after the T3 treatment. Treatment with T3 led to the upregulation of 229 DEGs and the down-regulation of 221 DEGs in cones. These data are consistent with the previous findings on the profound/broad roles of TH signaling in cones, ranging from the expression and retinal localization/distribution of cone opsins [29,41] to cone viability [30,37,38], and they support the view that cones are more susceptible to the stimulation of TH signaling among retinal cells. In contrast, the rods responded moderately to the T3 treatment, with 51 DEGs accounting for 4.4% of the total DEGs. Of note, retinal glial cells showed great responses to the T3 treatment, with 179 DEGs in Müller cells, 163 DEGs in astrocytes, and 100 DEGs in microglia, accounting for 15.3%, 14.0%, and 8.6% of the total DEGs, respectively. These data are consistent with the previous findings showing the increased labeling of the glial fibrillary acidic protein, a marker of Müller cell activation, and the increased labeling of Iba1, a marker of microglial activation, in the retinas after the T3 treatment [37,42]. Interestingly, it was recently reported that TH signaling in Müller cells coordinate retinal intercellular communications during light/dark adaptation [43].

The top 25 down-regulated DEGs in the cone cluster include almost all of the genes involved in phototransduction. Furthermore, both the canonical pathway analysis and the pathway/network analysis revealed that phototransduction was the most significantly inhibited pathway after T3 treatment. It was previously shown that the stimulation of TH signaling induces the expression of *Opn1mw* (encoding M-opsin) [28,29,32] and inhibits the expression of *Opn1sw* (encoding S-opsin). This work shows that excessive TH signaling inhibits the expressions of both *Opn1mw* and *Opn1sw*. The discrepancy on the expression of *Opn1mw* might be contributed by the experimental condition variations, including the age of the mice when they received the T3 treatment and the dose/duration of the treatment. Similar to cones, the top 34 down-regulated DEGs in the rods include genes involved in phototransduction. The canonical pathway analysis showed that phototransduction was the most significantly inhibited pathway after T3 treatment. The data showing reduced cone and rod phototransduction are consistent with the findings showing that the stimulation of TH signaling with the T3 treatment or deletion of Dio3 reduces cone and rod light responses [37,38].

A pathway analysis revealed that the oxidative phosphorylation pathway was profoundly suppressed in the cone cluster after the T3 treatment. The IPA also suggested the dysfunction of the mitochondrion in the cone cluster. The mitochondrion is a known major target of T3 through both a genetic mechanism via TH receptors and a non-genomic mechanism via truncated TH receptors or direct actions [1,44,45]. The data from the transcriptomic analysis are consistent with the findings obtained from the mitochondrial respiration activity assays, showing that treatment with T3 reduces the basal respiration, ATP production, and maximal respiration in the retina compared with the untreated controls [42]. The mitochondrial stress/malfunction might have also been reflected by the increased oxidative stress responses in the retina after the T3 treatment, manifested as an increased expression of oxidative stress genes [42] and increased labeling of oxidative stress/damage markers [37]. It seems reasonable to believe that excessive TH signaling impairs oxidative phosphorylation and bioenergetics, which may result in the accumulation of unstable molecules or free radicals and oxidative stress. Indeed, treatment with an antioxidant reduces T3-induced cone degeneration and cellular stress responses [37].

It was well characterized that excessive TH signaling induces cone death. However, the mechanism(s) behind this process remains less understood. As mentioned above, oxidative stress may play a role in the progression of cell death. The scRNAseq and IPA revealed additional cellular stress/death pathways that might be involved in T3-induced cone death. This includes the EIF2 signaling pathway and CREB signaling pathway. EIF2 signaling integrates a diverse array of stress-related signals to regulate mRNA translation, which, in turn, regulates signaling processes such as ER stress, apoptosis, amino acid metabolism, and glucose uptake. Specifically, the increased expressions of *Atf3*, *Ddit3*, and *Xiap* suggest an involvement of EIF2-ATF3-related ER stress/apoptosis (Supplementary Table S6). CREB signaling regulates diverse cellular processes, including proliferation, survival, and differentiation. CREB signaling can be activated by cAMP-dependent protein kinase A (PKA), protein kinase C (PKC), calmodulin kinases (e.g., CaMKII and CAMK4), and ribosomal S6 kinase (RPS6KA1). Indeed, PKA signaling was upregulated in cones after the T3 treatment (Figure 3; Table 2). The regulation of TH signaling on CaMK4/CREB signaling was reported previously [46]. Furthermore, the IPA network analysis and the upstream regulator analysis also suggest cellular compromise and the activation of the cell death pathways. Thus, TH-induced cone death appears to involve multiple cellular events and signaling pathways. These pathways merit further investigation for their specific roles in the detrimental effects of excessive TH signaling.

A comparison of DEGs in cones and rods shows a high overlap rate (70%; 36 out of 51) between the two types of photoreceptors (Supplementary Figure S4A). Among the 36 shared DEGs, 34 were altered in the same direction, and 2 were in the opposite direction (Supplementary Figure S4A; Supplementary Table S10), suggesting that the rods responded to the T3 treatment similarly to the cones. Nevertheless, T3 induces many more DEGs and canonical pathway alterations in cones compared to those induced in rods, as analyzed by the Benjamani–Hochberg adjusted *p*-values (Supplementary Figure S4B). T3 induces oxidative phosphorylation, EIF2 signaling, and CREB signaling, specifically in cones, whereas phototransduction, cAMP-mediated signaling, tRNA splicing, and gaseous signaling are induced in both cones and rods (Supplementary Figure S4B). Furthermore, T3 alters many more upstream regulators in cones compared with those altered in rods based on the z-scores (Supplementary Figure S4C). Of note, *Nrl* and *Ifng* are oppositely regulated in cones and rods (Supplementary Figure S4C). Similarly, an analysis of diseases and functions shows many more cellular events in cones compared with those in rods (Supplementary Figure S4D). Nevertheless, retinal degeneration, the degeneration of photoreceptors, organ degeneration, the neurodegeneration of sensory neurons, and neurodegeneration are among the top five upregulated cellular events in both cones and rods after T3 treatment (Supplementary Figure S4D).

This work, using a mouse model of hyperthyroidism, shows that excessive TH signaling suppresses phototransduction and mitochondrial bioenergetics at the transcriptomic levels and induces cellular stress/death pathways. Whether these cellular events are altered at the protein levels remains to be determined. Also, the way in which the genes encoding the functional proteins are regulated by TH signaling remains to be investigated. Moreover, whether the observations of mice mimic the cellular events/pathogenesis in the human retina remains to be investigated. Clinical studies have shown that an elevated TH level in circulation is associated with an increased incidence of AMD. Thus, the findings from this animal-based work support future efforts to examine TH signaling activity in the retinas of mice models of AMD and in AMD donor eyes, in addition to its relevance to phototransduction, mitochondrial bioenergetics/function, and cellular stress. With the establishment of the contribution of TH signaling to AMD pathogenesis, inhibiting TH signaling locally in the retina, such as through the use of Dio2 inhibitors or TH receptor antagonists, might be a strategy for the management of AMD.

In summary, this work, using scRNAseq, shows that cone transcriptomes are at the top among retinal cells after T3 treatment. The IPA indicates that there was impaired phototransduction and mitochondrial bioenergetics after the T3 treatment, which are concomitant with the upregulation of the cellular stress/death pathways. These findings provide an insight into how excessive TH signaling induces cone degeneration.

## 4. Materials and Methods

### 4.1. Thyroid Hormone Treatment

C57BL/6J mice were obtained from the Jackson Laboratory and used in this study. Mice were maintained under cyclic light (12 h light–dark) conditions. Cage illumination was carried out using a 7-foot candle during the light cycle. All animal maintenance processes and experiments were approved by the local Institutional Animal Care and Use Committee (University of Oklahoma Health Sciences Center) and conformed to the guidelines on the care and use of animals adopted by the Society for Neuroscience and the Association for Research in Vision and Ophthalmology. Mice of both genders were used. T3 for the administration of drinking water was prepared as described [47]. An amount of 10 mg of T3 (T2877, Sigma-Aldrich, St. Louis, MO, USA) was dissolved in 1.0 mL of 1.0 N NaOH, followed by dilution with tap water to obtain the final working concentrations.

### 4.2. Retinal Dissection and Dissociation

Eyes from the control and treated mice were placed in Dulbecco’s Modified Eagle Medium media (ThermoFisher, Richardson, TX, USA) and dissected to obtain the retinas. One retina was snap-frozen in liquid nitrogen and stored at −80 °C for RNA isolation, and the other one was used for retinal cell dissociation following the instruction of a Neural Tissue Dissociate Kit-Postnatal Neurons (cat# 130-094-802, Miltenyi Biotec, Auburn, CA, USA). Briefly, the retinal samples were washed twice with DPBS with calcium and magnesium (cat# D8662, Sigma-Aldrich, St. Louis, MO, USA) supplemented with 0.5% BSA before adding 1960 µL of enzyme mix 1. Retinas were incubated in enzyme mix 1 for 15 min at 37 °C under slow rotation, followed by the addition of 30 µL of enzyme mix 2. Following gentle pipetting up and down, the retinas were incubated for another 10 min at 37 °C. Then, 15 µL of enzyme mix 2 was added, and retinal tissue was further dissociated by pipetting up and down and filtering through a 70 µm SmartStrainer (cat# 130-098-462, Miltenyi Biotec). The dissociated cells were centrifuged at 130× *g* for 10 min at room temperature. Following washing and filtering, the resulting single-cell suspensions free from debris and cell aggregates were obtained. Cell density was counted, and viability (cat# 130-093-233, propidium iodide) was analyzed by flow cytometry (cat# 130-096-343, MACSQuant Analyzer). Cells were then diluted to 1000 cells per microliter prior to loading on the single-cell platform to target the recovery of 5000 cells per sample during scRNA-seq encapsulation. To keep cells healthy and intact, cell suspensions were kept on ice while preparing to load chips and loaded slowly and gently using wide-bore pipette tips.

### 4.3. Library Construction and scRNA-Seq

scRNA-seq libraries were constructed with the Chromium Single Cell 3ʹ GEM, Library & Gel Bead Kit v3 (cat# 10000075, 10× Genomics, Pleasanton, CA, USA) according to the manufacturer’s instructions as described previously [48]. The diluted cells, master mix, gel beads, and partitioning oil were added to the Chromium Single Cell B Chip (cat# 10000073, 10× Genomics) and loaded into the Chromium Controller (cat# 1000204, 10× Genomics) to generate the gel beads-in-emulsion (GEMs) for downstream library preparation. GEMs were then transferred to PCR strip tubes and incubated in a thermocycler to perform reverse transcription in GEMs (GEM-RT). Following GEM-RT, the recovery agent was aspirated, and cDNA was cleaned using the Dynabeads MyOne SILANE reagent included in the scRNA-seq kit. The cDNA was amplified and then cleaned using SPRISelect reagent beads (cat# B23318, Beckman Coulter, Indianapolis, IN, USA). The cDNA was quality checked using a High Sensitivity D5000 ScreenTape (cat# 5067-5592, Agilent, Santa Clara, CA, USA) run on a TapeStation 2200 (cat#G2964AA, Agilent). An aliquot of 25% of amplified cDNA was carried forward for library preparation. Libraries were quantified by qPCR and quality checked on a High Sensitivity D1000 ScreenTape (cat# 5067-5584, Agilent) on TapeStation 2220. Libraries were normalized, pooled, and sequenced using an Illumina NovaSeq 6000 system (SP PE50bp, S4 PE150). The sequence depth obtained was ~50,000 reads/cell.

### 4.4. Data Preprocessing and Statistics

Following sequencing, reads were trimmed and aligned, and differential expression statistics and correlation analyses were performed as described previously [48]. Reads were aligned against the Mm10 build of the mouse genome (2014.11.26). Cellranger mkfastq was used to demultiplex fastq files from raw base call (BCL) files. Cellranger_counts and Cellranger_aggregates were used with default settings. Cells with high mitochondrial counts were removed, and UMAP clustering was performed. To conduct a statistical analysis of differential expression, Student’s *t*-test was used to compare the differences between two groups, and significant differences were defined as *p* <  0.05. For transcripts meeting this statistical criterion, a fold change cutoff of >1.25 was used to eliminate those genes, which were statistically significant but unlikely to be biologically significant and orthogonally confirmable due to their very small magnitude of change.

### 4.5. Cell Characterization and Identification of Differentially Expressed Genes

Loupe Browser (10× Genomics) was used to characterize cell types. Cell type selection was based on the specific gene expression signature profiles in the UMAP and t-SNE plots [49,50,51] (See Table 1). Differentially expressed genes were obtained by the locally distinguishing comparison based on cell types after the T3 treatment at a *p*-value of 0.05.

### 4.6. Ingenuity Pathway Analysis

Ingenuity pathway analysis software (IPA, Qiagen, QIAGEN Digital Insights, Redwood City, CA, USA) was used to identify the most significantly affected canonical pathways, upstream regulators, and diseases and functions in the datasets based on the DEGs.

### 4.7. RNA Isolation and Quantitative Real-Time PC

Total RNA from retina corresponding to the individual mouse submitted for scRNAseq was isolated by following the PureLink^TM^ RNA Mini Kit (Ca# 12183018A, ThermoFisher, Richardson, TX, USA), and reverse transcription was performed using the iScript Reverse Transcription Supermix for qRT-PCR (Cat# 1708841, Bio-Rad, Hercules, CA, USA) as described previously [52]. The gene encoding the mouse hypoxanthine guanine phosphoribosyl transferase 1 (*Hprt1*) was included as an internal control. Supplementary Table S11 shows the primers used. The iTaq Universal SYBR^®^ Green Supermix (Cat# 1725121, Bio-Rad) was used for qRT-PCR assays in a Bio-Rad CFX Connect^TM^ Real-Time System, and the relative gene expression value was calculated based on the ΔΔCt method as described previously [52].

## Figures and Tables

**Table 1:** Cell types and DEGs in retinal cells after T3 treatment.

Cell Type	Cell Number (%)	DEGs (%)	Marker Genes
Rods	118381 (76.0)	51 (4.4)	*Rho*, *Cnga1*, *Pde6a*, *Ppef2*, *Nr2e3*
Cones	6463 (4.1)	450 (38.5)	*Arr3*, *Opn1mw*, *Gnat2*, *Pde6h*
Ganglion cells	888 (0.6)	101 (8.6)	*Slc17a6*, *Nefm*, *Cncg*, *Nefl*
Bipolar cells	14531 (9.3)	96 (8.2)	*Camk2b*, *Trpm1*, *Tmem215*, *Grm6*
Amacrine cells	2185 (1.4)	10 (0.9)	*Gad1*, *C1ql2*, *Calb1*, *Tmem215*
Müller cells	7716 (5.0)	179 (15.3)	*Clue*, *Glul*, *Apoe*, *Rlbp1*, *Crabp1*
Horizontal cells	979 (0.6)	10 (0.9)	*Lhx1*, *Onecut1*, *Onecut2*, *Calb1*
Microglia	808 (0.5)	100 (8.6)	*C1qa*, *Tmem119*, *Aif1*, *Cd163*, *Apoe*
Astrocytes	2959 (1.9)	163 (14.0)	*Gfap*, *Apoe*
RPE cells	138 (0.1)	5 (0.4)	*Rlbp1*, *Apoe*, *Crabp1*, *Trpm1*
Pericytes	184 (0.1)	4 (0.3)	*Acta2*, *Angpt2*
Vascular cells	30 (0.02)		*CD34*, *Adamts9*, *Rgs5*, *Cdh5*
Unsigned	604 (0.4)		
Total cells	155866 (100)		

**Table 2:** Canonical pathways identified from IPA.

Ingenuity Canonical Pathways	−log(*p*-Value)	z-Score
Phototransduction pathway	28.8	
Oxidative phosphorylation	6.93	−3.6
Mitochondrial dysfunction	5.79	3.3
Insulin secretion signaling pathway	3.82	3.5
Dopamine-DARPP32 feedback in cAMP signaling	5.08	2.9
CREB signaling in neurons	7.06	2.2
Sirtuin signaling pathway	4.53	2.1
Glutamate receptor signaling	10.9	0.3
Protein kinase A signaling	11.2	0.8
Signaling by Rho family GTPases	4.47	1.3
EIF2 Signaling	13	1.4
S100 family signaling pathway	3.69	2.0
Gustation pathway	3.44	2.1
Chemokine signaling	3.95	2.1
Renin-angiotensin signaling	2.77	2.2
Prolactin signaling	2.11	2.2
Granzyme A signaling	2.61	2.4

## Data Availability

The data generated and analyzed during the current study are available from the corresponding author upon reasonable request.

## Supplementary Materials

The supporting information can be downloaded at https://www.mdpi.com/article/10.3390/ijms25137435/s1.
