# Supplementary material for: Transcriptomic Analysis Reveals That Excessive Thyroid Hormone Signaling Impairs Phototransduction and Mitochondrial Bioenergetics and Induces Cellular Stress in Mouse Cone Photoreceptors
Source: Int J Mol Sci. 2024 Jul 6;25(13):7435. doi: 10.3390/ijms25137435 (PMC11242393; doi:10.3390/ijms25137435)
Supplement: Supplementary file 1 [file ijms-25-07435-s001.zip › ijms-3056379-supplementary.pdf]

For consideration of publication in International Journal of Molecular Sciences

Transcriptomic Analysis Reveals That Excessive Thyroid Hormone Signaling Impairs Phototransduction and Mitochondrial Bioenergetics and Induces Cellular Stress in Mouse Cone Photoreceptors

Hongwei Ma[1], David Stanford[2], Willard M. Freeman[2], and Xi-Qin Ding[1*]

[1]Department of Cell Biology, University of Oklahoma Health Sciences Center, Oklahoma City, Oklahoma, [2]Genes & Human Disease Research Program, Oklahoma Medical Research Foundation, Oklahoma City, Oklahoma

Supplementary Information

(Supplementary Figures S1-S4, Supplementary Tables S1-S11)

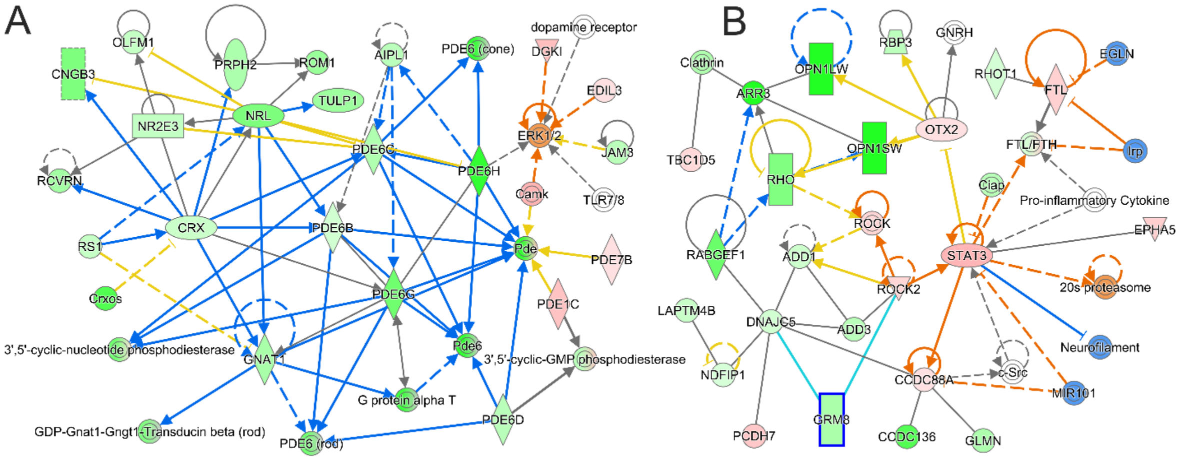

Supplementary Figure S1. Prominent pathway networks in cones after T3 treatment. **A.** Shown are the pathway networks related to developmental disorder, hereditary disorder, and ophthalmic disease in cones after T3 treatment. **B.** Shown are the pathway networks related to cell death/survival, cellular compromise, and neurological diseases in cones after T3 treatment.

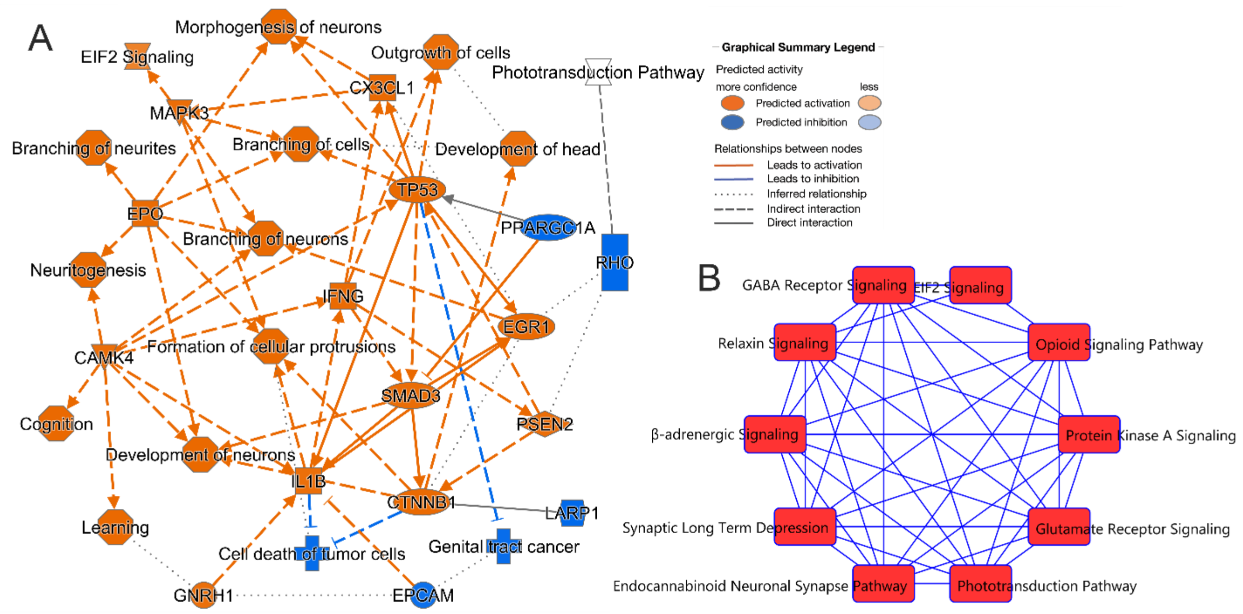

Supplementary Figure S2. Graphic summary and network analysis in cones after T3 treatment revealed by IPA. **A.** Shown is a graphic summary of gene networks altered in cones after T3 treatment. **B.** Overlapping of the top 10 canonical pathways in cones after T3 treatment.

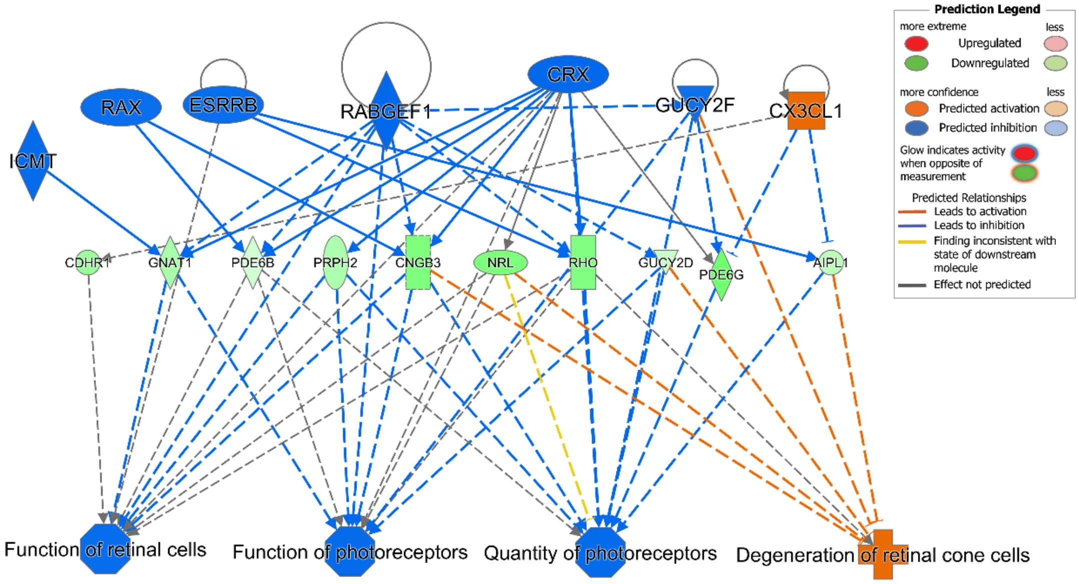

Supplementary Figure S3. Upstream regulators in cones after T3 treatment. Shown are the upstream regulators that are involved in gene expression, functions, and cell degeneration in cones after T3 treatment.

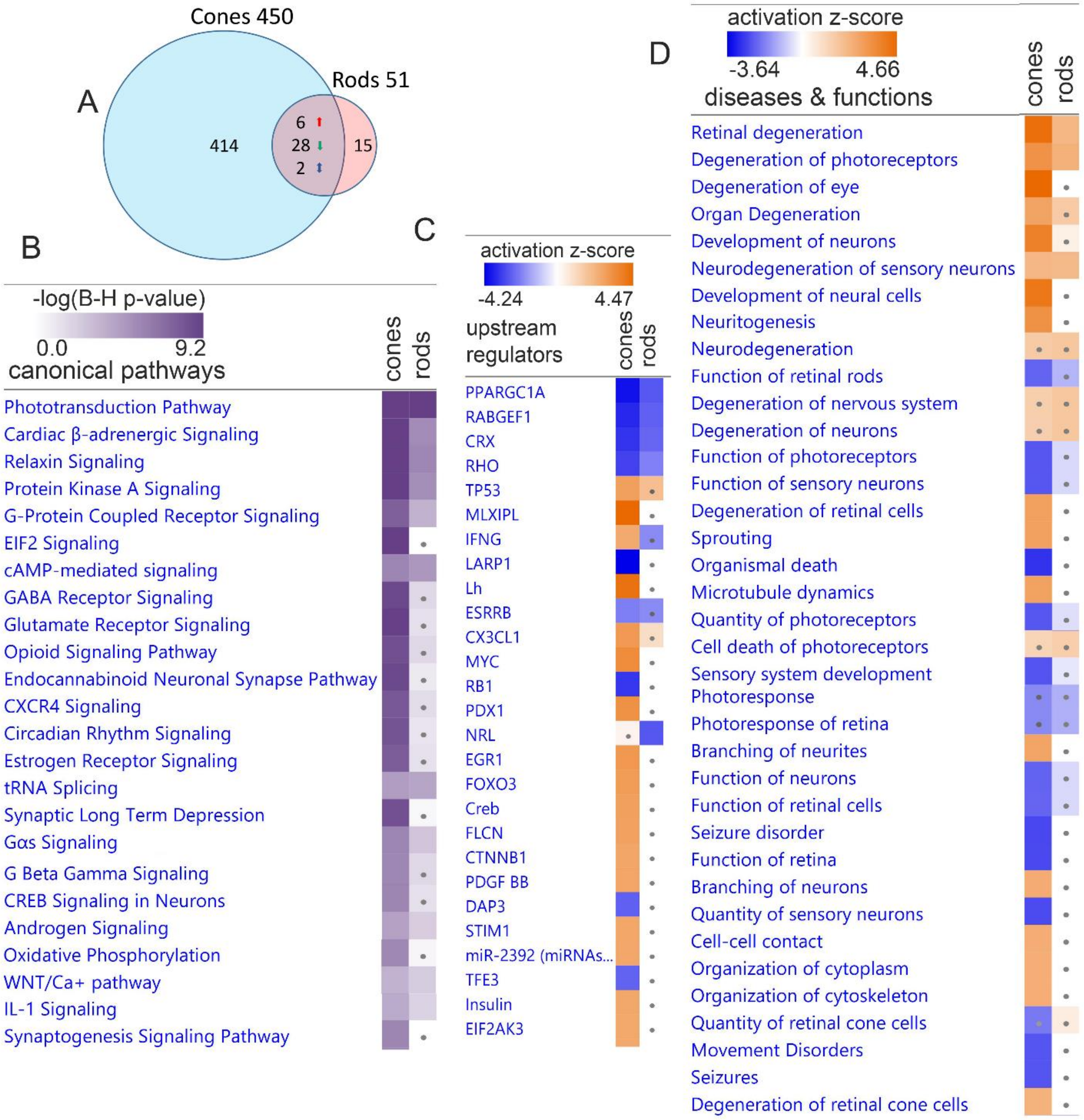

Supplementary Figure S4. Comparison of T3-induced transcriptomic alterations between cones and rods. **A.** A diagram showing 36 of the 51 DEGs in rods shared with cones, with 34 expressed in the same direction and 2 in the opposite direction. **B.** Shared canonical pathways in cones and rods after T3 treatment [Dots indicate -log(B-H p-value)<2]. **C.** Comparison of the upstream regulators in cones and rods after T3 treatment based on activation z-score [Dots indicate z-score

<2]. **D.** Comparison of diseases and functions in cones and rods after T3 treatment based on activation z-score [Dots indicate z-score <2].

Supplementary Table S1. DEGs in retinal cells after T3 treatment.

Feature ID	Gene name	Average expression level in control	Average expression in T3 treatment	Log2FC	P-Value
ENSMUSG00000086503	Xist	0.000	1.924	13.40	0.00E+00
ENSMUSG00000058831	Opn1sw	1.174	0.040	-4.88	9.32E-126
ENSMUSG00000064330	Pde6h	1.444	0.060	-4.59	7.39E-116
ENSMUSG00000031762	Mt2	0.060	1.293	4.43	4.42E-90
ENSMUSG00000031765	Mt1	0.313	4.208	3.75	1.65E-69
ENSMUSG00000040632	Nrl	1.477	0.230	-2.68	1.39E-46
ENSMUSG00000029064	Gnb1	4.034	0.697	-2.53	5.99E-42
ENSMUSG00000022037	Clu	0.450	2.770	2.62	2.89E-38
ENSMUSG00000030324	Rho	15.366	3.070	-2.32	9.07E-36
ENSMUSG00000025386	Pde6g	3.758	0.832	-2.17	1.34E-31
ENSMUSG00000024227	Pdzph1	1.542	0.344	-2.16	2.92E-31
ENSMUSG00000075410	Prcd	1.044	0.253	-2.05	3.31E-28
ENSMUSG00000071648	Rom1	4.080	1.092	-1.90	1.42E-24
ENSMUSG00000006007	Pdc	5.636	1.600	-1.82	1.42E-22
ENSMUSG00000023979	Guca1b	2.039	0.597	-1.77	1.46E-21
ENSMUSG00000034837	Gnat1	3.648	1.081	-1.75	3.71E-21
ENSMUSG00000032292	Nr2e3	1.106	0.353	-1.65	8.56E-19
ENSMUSG00000057132	Rpgrip1	2.008	0.642	-1.64	1.01E-18
ENSMUSG00000029663	Gngt1	7.721	2.587	-1.58	2.55E-17
ENSMUSG00000002985	Apoe	1.569	5.002	1.67	2.64E-17
ENSMUSG00000000628	Hk2	1.148	0.389	-1.56	5.45E-17
ENSMUSG00000002058	Unc119	4.134	1.491	-1.47	3.33E-15
ENSMUSG00000029491	Pde6b	1.561	0.568	-1.46	6.31E-15
ENSMUSG00000023978	Prph2	4.523	1.659	-1.45	9.90E-15
ENSMUSG00000001211	Agpat3	1.268	0.503	-1.33	1.13E-12
ENSMUSG00000052837	Junb	0.507	1.306	1.37	4.04E-12
ENSMUSG00000019978	Epb41l2	1.289	0.526	-1.29	6.21E-12
ENSMUSG00000091537	Tma7	4.359	1.784	-1.29	6.99E-12
ENSMUSG00000061080	Lsamp	0.859	2.194	1.35	7.01E-12
ENSMUSG00000028125	Abca4	1.004	0.415	-1.27	1.23E-11
ENSMUSG00000037446	Tulp1	1.941	0.818	-1.25	3.50E-11
ENSMUSG00000021250	Fos	0.855	2.101	1.30	4.45E-11
ENSMUSG00000050556	Kcnb1	1.397	0.610	-1.19	2.53E-10
ENSMUSG00000031789	Cngb1	1.086	0.481	-1.17	5.05E-10
ENSMUSG00000024575	Pde6a	1.851	0.821	-1.17	5.44E-10
ENSMUSG00000029088	Kcnip4	0.791	1.855	1.23	6.32E-10

ENSMUSG00000042757	Tmem108	1.082	0.482	-1.17	6.50E-10
ENSMUSG00000085794	Vax2os	1.133	0.511	-1.15	1.23E-09
ENSMUSG00000052684	Jun	0.743	1.697	1.19	1.59E-09
ENSMUSG00000030096	Slc6a6	1.519	0.709	-1.10	7.07E-09
ENSMUSG00000071076	Jund	0.539	1.176	1.13	1.22E-08
ENSMUSG00000055421	Pcdh9	0.913	1.993	1.13	1.32E-08
ENSMUSG00000024519	Cplx4	1.047	0.506	-1.05	3.58E-08
ENSMUSG00000052920	Prkg1	0.715	1.521	1.09	3.91E-08
ENSMUSG00000032087	Dscaml1	1.097	0.533	-1.04	4.68E-08
ENSMUSG00000034452	Slc24a1	1.008	0.491	-1.04	5.26E-08
ENSMUSG00000034912	Mdga2	0.954	1.965	1.04	1.58E-07
ENSMUSG00000067220	Cnga1	1.710	0.856	-1.00	1.80E-07
ENSMUSG00000020907	Rcvrn	3.041	1.530	-0.99	2.19E-07
ENSMUSG00000058966	Fam57b	1.088	0.553	-0.98	3.35E-07
ENSMUSG00000028399	Ptprd	0.714	1.414	0.99	7.31E-07
ENSMUSG00000028524	Sgip1	1.666	0.866	-0.94	8.89E-07
ENSMUSG00000092094	Zfp804b	1.713	3.366	0.97	1.06E-06
ENSMUSG00000002459	Rgs20	1.386	0.727	-0.93	1.27E-06
ENSMUSG00000054423	Cadps	0.651	1.228	0.92	4.61E-06
ENSMUSG00000023982	Guca1a	2.722	1.474	-0.88	4.62E-06
ENSMUSG00000066392	Nrxn3	2.856	5.321	0.90	7.18E-06
ENSMUSG00000029580	Actb	0.628	1.151	0.87	1.25E-05
ENSMUSG00000029673	Auts2	0.694	1.270	0.87	1.35E-05
ENSMUSG00000036469	1-Mar	2.999	1.672	-0.84	1.41E-05
ENSMUSG00000041534	Rbp3	1.058	0.597	-0.83	2.13E-05
ENSMUSG00000057914	Cacnb2	1.809	1.020	-0.83	2.16E-05
ENSMUSG00000022708	Zbtb20	1.239	2.232	0.85	2.29E-05
ENSMUSG00000056158	Car10	0.657	1.174	0.84	3.18E-05
ENSMUSG00000023439	Gnb3	0.890	1.577	0.83	3.96E-05
ENSMUSG00000069601	Ank3	0.770	1.357	0.82	4.85E-05
ENSMUSG00000071866	Ppia	0.628	1.097	0.80	6.65E-05
ENSMUSG00000033569	Adgrb3	0.669	1.135	0.76	1.59E-04
ENSMUSG00000008348	Ubc	0.715	1.212	0.76	1.63E-04
ENSMUSG00000027674	Pex5l	1.806	1.082	-0.74	1.70E-04
ENSMUSG00000031293	Rs1	1.111	0.665	-0.74	1.71E-04
ENSMUSG00000045103	Dmd	4.833	2.917	-0.73	2.18E-04
ENSMUSG00000063229	Ldha	1.094	0.668	-0.71	3.19E-04
ENSMUSG00000022748	Cmss1	6.653	4.106	-0.70	4.42E-04
ENSMUSG00000059974	Ntm	1.170	0.729	-0.68	5.83E-04
ENSMUSG00000098178	Gm42418	26.444	16.481	-0.68	5.96E-04
ENSMUSG00000028906	Epb41	1.237	0.776	-0.67	7.17E-04

ENSMUSG00000029632	Ndufa4	1.473	0.930	-0.66	8.96E-04
ENSMUSG00000032294	Pkm	2.171	1.374	-0.66	9.51E-04
ENSMUSG00000040003	Magi2	1.815	2.895	0.67	1.01E-03
ENSMUSG00000024500	Ppp2r2b	2.058	1.328	-0.63	1.68E-03
ENSMUSG00000030064	Frmd4b	1.158	0.747	-0.63	1.68E-03
ENSMUSG00000052613	Pcdh15	3.008	1.978	-0.60	2.82E-03
ENSMUSG00000047888	Tnrc6b	1.002	0.662	-0.60	3.23E-03
ENSMUSG00000032238	Rora	1.215	1.851	0.61	3.52E-03
ENSMUSG00000100252	Mir124-2hg	1.796	1.193	-0.59	3.67E-03
ENSMUSG00000025900	Rp1	2.117	1.425	-0.57	5.23E-03
ENSMUSG00000029635	Cdk8	1.370	0.932	-0.56	6.78E-03
ENSMUSG00000022108	Itm2b	0.747	1.104	0.56	7.46E-03
ENSMUSG00000037742	Eef1a1	1.141	1.681	0.56	7.92E-03
ENSMUSG00000022961	Son	1.436	0.986	-0.54	8.51E-03
ENSMUSG00000033676	Gabrb3	0.841	1.230	0.55	9.63E-03
ENSMUSG00000052727	Map1b	1.039	0.739	-0.49	1.93E-02
ENSMUSG00000023175	Bsg	1.068	1.504	0.49	2.17E-02
ENSMUSG00000035242	Oaz1	1.845	1.323	-0.48	2.32E-02
ENSMUSG00000025739	Gng13	0.717	1.002	0.48	2.57E-02
ENSMUSG00000037161	Mgarp	2.185	3.026	0.47	3.07E-02
ENSMUSG00000029086	Prom1	1.094	0.799	-0.45	3.35E-02
ENSMUSG00000068205	Macrod2	1.272	0.933	-0.45	3.70E-02
ENSMUSG00000030695	Aldoa	2.537	1.868	-0.44	4.02E-02
ENSMUSG00000031633	Slc25a4	0.947	1.285	0.44	4.52E-02
ENSMUSG00000057666	Gapdh	2.377	1.782	-0.42	5.64E-02
ENSMUSG00000020719	Ddx5	1.723	1.304	-0.40	6.71E-02
ENSMUSG00000026987	Baz2b	1.033	0.782	-0.40	6.76E-02
ENSMUSG00000027447	Cst3	1.785	1.351	-0.40	6.77E-02
ENSMUSG00000041841	Rpl37	0.760	1.006	0.41	7.06E-02
ENSMUSG00000037386	Rims2	1.859	1.413	-0.40	7.29E-02
ENSMUSG00000063077	Kif1b	1.351	1.036	-0.38	8.45E-02
ENSMUSG00000039145	Camk1d	1.207	1.581	0.39	8.63E-02
ENSMUSG00000004630	Pcp2	0.794	1.037	0.38	9.24E-02
ENSMUSG00000026609	Ush2a	1.213	0.937	-0.37	9.59E-02
ENSMUSG00000057322	Rpl38	0.815	1.054	0.37	1.06E-01
ENSMUSG00000021730	Hcn1	2.807	2.195	-0.35	1.18E-01
ENSMUSG00000040554	Aipl1	1.096	0.859	-0.35	1.21E-01
ENSMUSG00000053819	Camk2d	1.563	1.246	-0.33	1.58E-01
ENSMUSG00000060743	H3f3a	1.593	2.007	0.33	1.58E-01
ENSMUSG00000060636	Rpl35a	0.822	1.034	0.33	1.62E-01
ENSMUSG00000064358	mt-Co3	16.061	12.825	-0.32	1.63E-01

ENSMUSG00000093674	Rpl41	0.918	1.144	0.32	1.85E-01
ENSMUSG00000039001	Rps21	0.933	1.154	0.31	2.04E-01
ENSMUSG00000098682	Otx2os1	1.632	2.018	0.31	2.04E-01
ENSMUSG00000105361	AY036118	1.026	0.836	-0.30	2.15E-01
ENSMUSG00000000740	Rpl13	1.307	1.600	0.29	2.32E-01
ENSMUSG00000001175	Calm1	1.753	2.137	0.29	2.47E-01
ENSMUSG00000025393	Atp5b	1.017	0.841	-0.27	2.61E-01
ENSMUSG00000064370	mt-Cytb	11.967	9.906	-0.27	2.65E-01
ENSMUSG00000064360	mt-Nd3	2.191	2.643	0.27	2.80E-01
ENSMUSG00000071415	Rpl23	1.063	1.281	0.27	2.83E-01
ENSMUSG00000032826	Ank2	0.861	1.031	0.26	3.04E-01
ENSMUSG00000035530	Eif1	1.556	1.863	0.26	3.07E-01
ENSMUSG00000020460	Rps27a	1.219	1.443	0.24	3.49E-01
ENSMUSG00000064367	mt-Nd5	1.282	1.088	-0.24	3.57E-01
ENSMUSG00000056055	Sag	7.275	6.190	-0.23	3.63E-01
ENSMUSG00000064341	mt-Nd1	2.817	3.319	0.24	3.63E-01
ENSMUSG00000037266	Rsrp1	1.168	0.994	-0.23	3.65E-01
ENSMUSG00000026473	Glul	0.894	1.052	0.23	3.70E-01
ENSMUSG00000075706	Gpx4	1.345	1.152	-0.22	3.87E-01
ENSMUSG00000047454	Gphn	1.330	1.140	-0.22	3.93E-01
ENSMUSG00000060126	Tpt1	1.656	1.936	0.23	3.93E-01
ENSMUSG00000092341	Malat1	69.390	80.879	0.22	4.04E-01
ENSMUSG00000047215	Rpl9	1.413	1.218	-0.21	4.13E-01
ENSMUSG00000064357	mt-Atp6	20.867	18.044	-0.21	4.27E-01
ENSMUSG00000021270	Hsp90aa1	2.991	2.587	-0.21	4.27E-01
ENSMUSG00000046330	Rpl37a	0.902	1.038	0.20	4.60E-01
ENSMUSG00000046364	Rpl27a	0.918	1.053	0.20	4.74E-01
ENSMUSG00000004637	Wwox	1.244	1.092	-0.19	4.90E-01

Supplementary Table S2. DEGs in cones after T3 treatment.

Feature ID	Gene name	Average expression level in control	Average expression level in T3 treatment	Log2 FC	P-Value
ENSMUSG00000086503	Xist	0.000	9.378	15.46	1.39E-274
ENSMUSG00000031762	Mt2	0.043	2.129	5.62	2.23E-97
ENSMUSG00000031765	Mt1	0.227	6.772	4.9	3.25E-82
ENSMUSG00000071637	Cebpd	0.069	1.391	4.34	5.40E-67
ENSMUSG00000032495	Lrrc2	0.069	1.298	4.23	4.53E-65
ENSMUSG00000026628	Atf3	0.065	1.097	4.07	1.68E-57
ENSMUSG00000001025	S100a6	0.073	1.153	3.98	2.70E-54
ENSMUSG00000052837	Junb	0.225	2.054	3.19	3.36E-41
ENSMUSG00000025351	Cd63	0.274	1.948	2.83	7.65E-34
ENSMUSG00000002985	Apoe	0.809	5.673	2.81	9.35E-31
ENSMUSG00000052684	Jun	0.522	3.052	2.55	7.91E-28
ENSMUSG00000040896	Kcnd3	0.361	2.076	2.52	5.04E-26
ENSMUSG00000038418	Egr1	0.312	1.658	2.41	2.87E-24
ENSMUSG00000051401	Kctd16	0.341	1.791	2.39	1.20E-23
ENSMUSG00000052387	Trpm3	0.974	4.920	2.34	8.35E-24
ENSMUSG00000056296	Synpr	0.206	1.024	2.31	1.74E-20
ENSMUSG00000008575	Nfib	1.201	5.867	2.29	1.14E-22
ENSMUSG00000029088	Kcnip4	12.115	58.499	2.27	1.58E-22
ENSMUSG00000066113	Adamtsl1	1.450	6.977	2.27	2.89E-22
ENSMUSG00000040118	Cacna2d1	1.104	5.212	2.24	6.65E-22
ENSMUSG00000022037	Clu	0.843	3.919	2.22	3.46E-21
ENSMUSG00000090223	Pcp4	1.703	7.881	2.21	2.46E-21
ENSMUSG00000021250	Fos	0.769	3.542	2.2	6.75E-21
ENSMUSG00000042846	Lrrtm3	0.463	2.111	2.19	2.85E-20
ENSMUSG00000060843	Ctnna3	0.230	1.037	2.17	1.23E-19
ENSMUSG00000031673	Cdh11	0.527	2.356	2.16	2.97E-20
ENSMUSG00000037605	Adgrl3	0.339	1.478	2.12	1.98E-19
ENSMUSG00000044071	Fam19a2	0.478	2.076	2.12	4.78E-19
ENSMUSG00000000263	Glra1	0.328	1.384	2.08	1.61E-18
ENSMUSG00000063887	Nlgn1	1.243	5.232	2.07	5.93E-19
ENSMUSG00000063600	Egfem1	0.620	2.611	2.07	1.80E-18
ENSMUSG00000052920	Prkg1	0.273	1.114	2.03	2.00E-14
ENSMUSG00000034780	B3galt1	0.439	1.778	2.02	1.20E-17
ENSMUSG00000063873	Slc24a3	1.108	4.353	1.97	3.67E-17
ENSMUSG00000046159	Chrm3	1.331	5.176	1.96	6.57E-17
ENSMUSG00000051910	Sox6	0.456	1.747	1.94	2.88E-16

ENSMUSG00000073565	Prr16	0.288	1.084	1.91	3.69E-15
ENSMUSG00000049690	Nckap5	0.489	1.820	1.9	8.90E-16
ENSMUSG00000061080	Lsamp	1.593	5.930	1.9	9.86E-16
ENSMUSG00000034310	Tmem132d	0.440	1.638	1.9	1.85E-15
ENSMUSG00000022935	Grik1	8.852	32.628	1.88	8.96E-16
ENSMUSG00000028565	Nfia	0.452	1.668	1.88	2.58E-15
ENSMUSG00000016918	Sulf1	0.335	1.218	1.86	9.14E-15
ENSMUSG00000039706	Ldb2	0.325	1.169	1.85	1.07E-14
ENSMUSG00000020524	Gria1	0.316	1.135	1.85	1.32E-14
ENSMUSG00000020627	Klhl29	0.372	1.328	1.83	8.97E-15
ENSMUSG00000026872	Zeb2	0.535	1.903	1.83	1.09E-14
ENSMUSG00000064293	Cntn4	2.288	8.094	1.82	8.66E-15
ENSMUSG00000003279	Dlgap1	0.297	1.034	1.8	1.61E-12
ENSMUSG00000070695	Cntnap5a	1.841	6.385	1.79	2.77E-14
ENSMUSG00000038128	Camk4	0.474	1.640	1.79	5.58E-14
ENSMUSG00000004040	Stat3	0.382	1.299	1.76	6.77E-14
ENSMUSG00000006204	5430419D17Rik	0.600	2.037	1.76	6.92E-14
ENSMUSG00000039539	Sgcz	0.365	1.200	1.72	1.07E-11
ENSMUSG00000033863	Klf9	0.631	2.063	1.71	2.80E-13
ENSMUSG00000050587	Lrrc4c	2.092	6.819	1.7	5.96E-13
ENSMUSG00000027416	Otor	0.379	1.235	1.7	2.22E-12
ENSMUSG00000062151	Unc13c	1.041	3.370	1.69	1.15E-12
ENSMUSG00000067786	Nnat	0.536	1.710	1.67	3.78E-12
ENSMUSG00000028832	Stmn1	0.768	2.406	1.65	3.55E-12
ENSMUSG00000027784	Ppm1l	0.469	1.454	1.63	7.22E-12
ENSMUSG00000010095	Slc3a2	0.395	1.209	1.61	1.46E-11
ENSMUSG00000036019	Tmtc2	0.643	1.968	1.61	1.50E-11
ENSMUSG00000037940	Inpp4b	1.231	3.731	1.6	2.26E-11
ENSMUSG00000031284	Pak3	0.489	1.476	1.59	3.32E-11
ENSMUSG00000032036	Kirrel3	1.338	4.041	1.59	4.66E-11
ENSMUSG00000041078	Grid1	0.445	1.335	1.58	4.90E-11
ENSMUSG00000052889	Prkcb	0.433	1.276	1.56	9.24E-11
ENSMUSG00000015501	Hivep2	0.439	1.288	1.55	6.89E-11
ENSMUSG00000052504	Epha3	1.506	4.403	1.55	1.11E-10
ENSMUSG00000038665	Dgki	0.342	1.005	1.55	1.27E-10
ENSMUSG00000071076	Jund	0.996	2.900	1.54	6.10E-11
ENSMUSG00000025408	Ddit3	0.451	1.314	1.54	1.52E-10
ENSMUSG00000047842	Diras2	0.408	1.179	1.53	1.56E-10
ENSMUSG00000040797	Iqsec3	0.435	1.249	1.52	2.21E-10
ENSMUSG00000021239	Vsx2	0.581	1.666	1.52	2.25E-10
ENSMUSG00000049122	Frmd3	0.749	2.142	1.52	3.20E-10

ENSMUSG00000021477	Ctsl	0.521	1.471	1.5	3.06E-10
ENSMUSG00000063063	Ctnna2	1.565	4.433	1.5	3.43E-10
ENSMUSG00000026473	Glul	0.662	1.870	1.5	5.69E-10
ENSMUSG00000015968	Cacna1d	0.566	1.584	1.48	7.40E-10
ENSMUSG00000075256	Cerkl	0.791	2.187	1.47	7.97E-10
ENSMUSG00000022472	Desi1	0.792	2.160	1.45	1.04E-09
ENSMUSG00000037747	Phyhipl	0.675	1.843	1.45	1.49E-09
ENSMUSG00000051951	Xkr4	0.378	1.035	1.45	3.93E-09
ENSMUSG00000029673	Auts2	1.899	5.155	1.44	1.71E-09
ENSMUSG00000004347	Pde1c	0.745	2.001	1.42	3.30E-09
ENSMUSG00000055022	Cntn1	1.041	2.745	1.4	6.01E-09
ENSMUSG00000079157	Fam155a	1.596	4.144	1.38	1.53E-08
ENSMUSG00000022892	App	0.722	1.865	1.37	1.36E-08
ENSMUSG00000026657	Frmd4a	1.185	3.057	1.37	1.64E-08
ENSMUSG00000022332	Khdrbs3	1.502	3.793	1.34	2.58E-08
ENSMUSG00000056158	Car10	2.394	6.068	1.34	3.11E-08
ENSMUSG00000033910	Gucy1a1	0.487	1.230	1.34	4.45E-08
ENSMUSG00000063142	Kcnma1	2.764	6.948	1.33	3.15E-08
ENSMUSG00000031561	Tenm3	2.773	6.902	1.32	5.60E-08
ENSMUSG00000029108	Pcdh7	0.926	2.318	1.32	7.83E-08
ENSMUSG00000022876	Samsn1	2.133	5.206	1.29	1.24E-07
ENSMUSG00000068748	Ptprz1	0.696	1.707	1.29	1.34E-07
ENSMUSG00000037593	BC030499	0.973	2.366	1.28	1.65E-07
ENSMUSG00000003949	Hlf	0.578	1.383	1.26	2.89E-07
ENSMUSG00000056755	Grm7	0.746	1.782	1.26	4.35E-07
ENSMUSG00000110344	Gm45716	0.899	2.144	1.25	2.34E-07
ENSMUSG00000031523	Dlc1	0.823	1.955	1.25	3.52E-07
ENSMUSG00000061576	Dpp6	0.539	1.287	1.25	4.15E-07
ENSMUSG00000052632	Asap2	1.035	2.447	1.24	4.18E-07
ENSMUSG00000055421	Pcdh9	5.475	12.818	1.23	5.67E-07
ENSMUSG00000057455	Rit2	0.784	1.831	1.22	7.15E-07
ENSMUSG00000048078	Tenm4	0.532	1.239	1.22	1.26E-06
ENSMUSG00000055639	Dach1	1.271	2.892	1.19	1.39E-06
ENSMUSG00000054976	Nyap2	0.770	1.749	1.18	1.94E-06
ENSMUSG00000048015	Neurod4	0.588	1.325	1.17	2.73E-06
ENSMUSG00000029245	Epha5	0.663	1.485	1.16	3.47E-06
ENSMUSG00000055447	Cd47	0.746	1.652	1.15	3.05E-06
ENSMUSG00000024112	Cacna1h	0.460	1.023	1.15	3.88E-06
ENSMUSG00000010803	Gabra1	0.510	1.132	1.15	4.96E-06
ENSMUSG00000066392	Nrxn3	16.189	35.607	1.14	3.74E-06
ENSMUSG00000021313	Ryr2	1.009	2.221	1.14	3.94E-06

ENSMUSG00000030206	Gsg1	2.582	5.695	1.14	4.44E-06
ENSMUSG00000022240	Ctnnd2	0.605	1.334	1.14	5.32E-06
ENSMUSG00000038349	Plcl1	0.970	2.119	1.13	5.28E-06
ENSMUSG00000027351	Spred1	0.461	1.009	1.13	6.27E-06
ENSMUSG00000039943	Plcb4	1.387	3.022	1.12	4.48E-06
ENSMUSG00000021557	Agtpbp1	1.198	2.569	1.1	8.16E-06
ENSMUSG00000056752	Dnah9	0.764	1.634	1.1	1.34E-05
ENSMUSG00000041670	Rims1	0.520	1.111	1.1	1.83E-05
ENSMUSG00000050708	Ftl1	1.500	3.193	1.09	9.83E-06
ENSMUSG00000059824	Dbp	0.733	1.558	1.09	1.19E-05
ENSMUSG00000086020	Gm12239	0.576	1.223	1.09	1.63E-05
ENSMUSG00000024268	Celf4	0.740	1.571	1.08	1.36E-05
ENSMUSG00000060534	Dcc	3.228	6.824	1.08	1.52E-05
ENSMUSG00000092094	Zfp804b	16.535	34.880	1.08	1.77E-05
ENSMUSG00000026385	Dbi	0.694	1.467	1.08	1.88E-05
ENSMUSG00000024044	Epb41l3	0.822	1.722	1.07	2.06E-05
ENSMUSG00000030518	Fam189a1	0.711	1.487	1.06	2.39E-05
ENSMUSG00000054423	Cadps	3.713	7.694	1.05	2.87E-05
ENSMUSG00000021536	Adcy2	0.601	1.224	1.03	5.46E-05
ENSMUSG00000034731	Dgkh	0.495	1.011	1.03	5.59E-05
ENSMUSG00000021337	Scgn	0.613	1.243	1.02	7.73E-05
ENSMUSG00000022708	Zbtb20	3.448	6.928	1.01	5.10E-05
ENSMUSG00000009470	Tnpo1	1.301	2.594	1	7.09E-05
ENSMUSG00000050272	Dscam	2.908	5.747	0.98	9.15E-05
ENSMUSG00000070683	Lactbl1	0.752	1.482	0.98	1.14E-04
ENSMUSG00000020580	Rock2	0.616	1.215	0.98	1.16E-04
ENSMUSG00000028399	Ptprd	2.493	4.934	0.98	1.31E-04
ENSMUSG00000033569	Adgrb3	3.481	6.811	0.97	1.25E-04
ENSMUSG00000040430	Pitpnc1	1.138	2.227	0.97	1.32E-04
ENSMUSG00000008682	Rpl10	0.916	1.794	0.97	1.33E-04
ENSMUSG00000020140	Lgr5	0.597	1.172	0.97	1.63E-04
ENSMUSG00000020023	Tmcc3	0.772	1.500	0.96	2.01E-04
ENSMUSG00000018567	Gabarap	0.725	1.402	0.95	1.93E-04
ENSMUSG00000017843	Ppp2r5c	1.124	2.161	0.94	2.11E-04
ENSMUSG00000026468	Lhx4	0.699	1.343	0.94	2.58E-04
ENSMUSG00000021282	Eif5	0.807	1.507	0.9	4.63E-04
ENSMUSG00000056899	Immp2l	1.130	2.108	0.9	5.01E-04
ENSMUSG00000032010	Usp2	0.719	1.330	0.89	5.93E-04
ENSMUSG00000023923	Tbc1d5	0.580	1.075	0.89	6.32E-04
ENSMUSG00000020680	Taf15	0.621	1.149	0.89	6.36E-04
ENSMUSG00000024587	Nars	0.611	1.103	0.85	1.17E-03

ENSMUSG00000045095	Magi1	0.664	1.198	0.85	1.19E-03
ENSMUSG00000056596	Trnp1	0.755	1.363	0.85	1.32E-03
ENSMUSG00000021268	Meg3	1.540	2.773	0.85	1.38E-03
ENSMUSG00000001627	Ifrd1	0.589	1.061	0.85	1.44E-03
ENSMUSG00000015937	H2afy	1.475	2.643	0.84	1.16E-03
ENSMUSG00000005649	Cabp5	1.245	2.227	0.84	1.66E-03
ENSMUSG00000047843	Bri3	0.707	1.256	0.83	1.62E-03
ENSMUSG00000026615	Eprs	0.571	1.018	0.83	1.64E-03
ENSMUSG00000025739	Gng13	1.031	1.835	0.83	1.86E-03
ENSMUSG00000044499	Hs3st5	0.590	1.046	0.83	2.49E-03
ENSMUSG00000023267	Gabrr2	0.574	1.008	0.81	2.65E-03
ENSMUSG00000061603	Akap6	0.943	1.641	0.8	2.64E-03
ENSMUSG00000031626	Sorbs2	0.905	1.578	0.8	2.67E-03
ENSMUSG00000000740	Rpl13	4.576	7.911	0.79	2.64E-03
ENSMUSG00000050856	Atp5k	1.796	3.103	0.79	2.76E-03
ENSMUSG00000008348	Ubc	1.881	3.243	0.79	2.85E-03
ENSMUSG00000022307	Oxr1	1.181	2.036	0.79	3.07E-03
ENSMUSG00000056486	Chn1	0.628	1.087	0.79	3.14E-03
ENSMUSG00000024812	Tjp2	0.653	1.129	0.79	3.17E-03
ENSMUSG00000037742	Eef1a1	4.451	7.656	0.78	2.96E-03
ENSMUSG00000098682	Otx2os1	7.481	12.823	0.78	3.16E-03
ENSMUSG00000033981	Gria2	0.789	1.360	0.78	3.72E-03
ENSMUSG00000050965	Prkca	0.946	1.621	0.78	4.00E-03
ENSMUSG00000024304	Cdh2	1.002	1.701	0.76	4.28E-03
ENSMUSG00000020814	Mxra7	0.788	1.337	0.76	4.48E-03
ENSMUSG00000030020	Prickle2	0.866	1.467	0.76	4.89E-03
ENSMUSG00000090733	Rps27	2.530	4.264	0.75	4.78E-03
ENSMUSG00000041841	Rpl37	2.665	4.441	0.74	5.91E-03
ENSMUSG00000034488	Edil3	0.736	1.230	0.74	6.89E-03
ENSMUSG00000028081	Rps3a1	2.538	4.203	0.73	6.79E-03
ENSMUSG00000109394	A230057D06Rik	2.272	3.761	0.73	7.54E-03
ENSMUSG00000074656	Eif2s2	0.906	1.493	0.72	8.27E-03
ENSMUSG00000067288	Rps28	2.297	3.764	0.71	8.45E-03
ENSMUSG00000025362	Rps26	1.145	1.873	0.71	9.08E-03
ENSMUSG00000060636	Rpl35a	2.829	4.604	0.7	9.63E-03
ENSMUSG00000062006	Rpl34	2.274	3.700	0.7	9.71E-03
ENSMUSG00000060938	Rpl26	3.001	4.873	0.7	1.01E-02
ENSMUSG00000070866	Zfp804a	1.142	1.854	0.7	1.23E-02
ENSMUSG00000049517	Rps23	2.414	3.885	0.69	1.21E-02
ENSMUSG00000060126	Tpt1	4.843	7.791	0.69	1.21E-02
ENSMUSG00000028525	Pde4b	0.838	1.348	0.69	1.54E-02

ENSMUSG00000038900	Rpl12	1.231	1.974	0.68	1.34E-02
ENSMUSG00000057322	Rpl38	2.735	4.369	0.68	1.36E-02
ENSMUSG00000058546	Rpl23a	0.834	1.336	0.68	1.43E-02
ENSMUSG00000040952	Rps19	1.893	3.016	0.67	1.50E-02
ENSMUSG00000019990	Pde7b	0.782	1.243	0.67	1.90E-02
ENSMUSG00000053477	Tcf4	1.525	2.417	0.66	1.64E-02
ENSMUSG00000061983	Rps12	1.622	2.571	0.66	1.66E-02
ENSMUSG00000039756	Dnttip2	0.645	1.014	0.65	2.11E-02
ENSMUSG00000021848	Otx2	1.136	1.777	0.65	2.13E-02
ENSMUSG00000024063	Lbh	0.719	1.128	0.65	2.16E-02
ENSMUSG00000039485	Tspyl4	0.650	1.017	0.64	2.29E-02
ENSMUSG00000023826	Park2	1.101	1.719	0.64	2.30E-02
ENSMUSG00000022311	Csmd3	1.105	1.725	0.64	3.15E-02
ENSMUSG00000071415	Rpl23	3.803	5.885	0.63	2.46E-02
ENSMUSG00000030738	Eif3c	0.864	1.327	0.62	3.04E-02
ENSMUSG00000033713	Foxn3	1.019	1.562	0.62	3.22E-02
ENSMUSG00000057841	Rpl32	2.413	3.694	0.61	3.03E-02
ENSMUSG00000032740	Ccdc88a	0.762	1.164	0.61	3.38E-02
ENSMUSG00000025290	Rps24	5.028	7.635	0.6	3.43E-02
ENSMUSG00000036452	Arhgap26	0.939	1.422	0.6	3.96E-02
ENSMUSG00000089809	Rasgef1b	0.755	1.141	0.6	4.15E-02
ENSMUSG00000021314	Amph	1.932	2.915	0.59	3.94E-02
ENSMUSG00000026131	Dst	1.017	1.533	0.59	4.12E-02
ENSMUSG00000038518	Jarid2	0.815	1.225	0.59	4.40E-02
ENSMUSG00000020460	Rps27a	4.371	6.552	0.58	4.23E-02
ENSMUSG00000037563	Rps16	2.714	4.061	0.58	4.39E-02
ENSMUSG00000033676	Gabrb3	9.004	13.452	0.58	4.52E-02
ENSMUSG00000020372	Rack1	1.376	2.055	0.58	4.68E-02
ENSMUSG00000045128	Rpl18a	2.477	3.683	0.57	4.90E-02
ENSMUSG00000024425	Ndfip1	1.485	0.970	-0.61	4.82E-02
ENSMUSG00000023010	Tmbim6	1.429	0.934	-0.61	4.90E-02
ENSMUSG00000025757	Hspa4l	1.688	1.104	-0.61	4.92E-02
ENSMUSG00000064354	mt-Co2	64.559	41.876	-0.62	4.15E-02
ENSMUSG00000006057	Atp5g1	2.713	1.767	-0.62	4.54E-02
ENSMUSG00000021098	4930447C04Rik	2.706	1.765	-0.62	4.71E-02
ENSMUSG00000023236	Scg5	2.656	1.716	-0.63	3.99E-02
ENSMUSG00000024661	Fth1	8.366	5.352	-0.64	3.37E-02
ENSMUSG00000042207	Kdm5b	1.048	0.673	-0.64	3.98E-02
ENSMUSG00000054580	Pla2r1	1.155	0.743	-0.64	4.14E-02
ENSMUSG00000032294	Pkm	9.054	5.773	-0.65	3.19E-02
ENSMUSG00000026609	Ush2a	5.427	3.467	-0.65	3.39E-02

ENSMUSG00000017686	Rhot1	1.792	1.144	-0.65	3.39E-02
ENSMUSG00000020022	Ndufa12	1.496	0.955	-0.65	3.41E-02
ENSMUSG00000019699	Akt3	1.681	1.061	-0.66	2.93E-02
ENSMUSG00000067367	Lyar	1.143	0.722	-0.66	3.03E-02
ENSMUSG00000064357	mt-Atp6	92.506	58.253	-0.67	2.64E-02
ENSMUSG00000030629	Zfand6	1.624	1.020	-0.67	2.66E-02
ENSMUSG00000049353	Rd3	1.097	0.690	-0.67	2.85E-02
ENSMUSG00000008206	Cers4	1.635	1.020	-0.68	2.39E-02
ENSMUSG00000000826	Dnajc5	1.109	0.694	-0.68	2.62E-02
ENSMUSG00000028397	Kdm4c	2.542	1.571	-0.69	2.03E-02
ENSMUSG00000029491	Pde6b	2.531	1.573	-0.69	2.30E-02
ENSMUSG00000064363	mt-Nd4	24.977	15.376	-0.7	1.83E-02
ENSMUSG00000032192	Gnb5	2.718	1.662	-0.71	1.68E-02
ENSMUSG00000026260	Ndufa10	1.316	0.806	-0.71	1.79E-02
ENSMUSG00000028412	Slc44a1	1.095	0.669	-0.71	1.82E-02
ENSMUSG00000031865	Dctn1	1.248	0.764	-0.71	1.83E-02
ENSMUSG00000064367	mt-Nd5	6.940	4.221	-0.72	1.51E-02
ENSMUSG00000063972	Nr6a1	1.193	0.726	-0.72	1.70E-02
ENSMUSG00000035885	Cox8a	8.493	5.112	-0.73	1.24E-02
ENSMUSG00000001270	Ckb	17.724	10.617	-0.74	1.14E-02
ENSMUSG00000037475	Thoc2	1.567	0.941	-0.74	1.26E-02
ENSMUSG00000028977	Casz1	2.133	1.281	-0.74	1.30E-02
ENSMUSG00000058704	Memo1	1.143	0.685	-0.74	1.31E-02
ENSMUSG00000041126	H2afv	1.561	0.926	-0.75	1.02E-02
ENSMUSG00000078247	Airn	1.163	0.692	-0.75	1.25E-02
ENSMUSG00000078851	Hist3h2a	1.858	1.095	-0.76	9.09E-03
ENSMUSG00000019943	Atp2b1	4.560	2.694	-0.76	9.20E-03
ENSMUSG00000022564	Grina	1.913	1.129	-0.76	9.34E-03
ENSMUSG00000052727	Map1b	5.338	3.162	-0.76	9.53E-03
ENSMUSG00000027530	Fabp12	2.517	1.488	-0.76	9.53E-03
ENSMUSG00000037032	Apbb1	1.406	0.831	-0.76	9.88E-03
ENSMUSG00000031142	Cacna1f	1.303	0.771	-0.76	1.01E-02
ENSMUSG00000027827	Kcnab1	1.112	0.656	-0.76	1.10E-02
ENSMUSG00000022016	Akap11	1.786	1.044	-0.77	7.97E-03
ENSMUSG00000021986	Amer2	1.099	0.643	-0.77	8.52E-03
ENSMUSG00000064358	mt-Co3	78.846	45.807	-0.78	6.67E-03
ENSMUSG00000035242	Oaz1	10.639	6.194	-0.78	6.81E-03
ENSMUSG00000029106	Add1	1.154	0.673	-0.78	8.00E-03
ENSMUSG00000025437	Usp33	1.041	0.605	-0.78	8.03E-03
ENSMUSG00000064351	mt-Co1	54.529	31.539	-0.79	6.12E-03
ENSMUSG00000032120	C2cd2l	1.126	0.650	-0.79	6.67E-03

ENSMUSG00000023495	Pcbp4	1.963	1.126	-0.8	5.57E-03
ENSMUSG00000029804	Herc3	1.255	0.718	-0.8	6.02E-03
ENSMUSG00000064370	mt-Cytb	58.467	33.281	-0.81	4.59E-03
ENSMUSG00000027447	Cst3	5.373	3.065	-0.81	4.82E-03
ENSMUSG00000035270	Impg2	1.678	0.955	-0.81	5.02E-03
ENSMUSG00000041578	Crx	2.351	1.342	-0.81	5.07E-03
ENSMUSG00000020321	Mdh1	3.161	1.808	-0.81	5.11E-03
ENSMUSG00000103428	Gm20754	1.583	0.905	-0.81	1.21E-02
ENSMUSG00000063229	Ldha	4.499	2.548	-0.82	4.16E-03
ENSMUSG00000020069	Hnrnph3	1.971	1.120	-0.82	4.59E-03
ENSMUSG00000036158	Prickle1	1.382	0.783	-0.82	4.75E-03
ENSMUSG00000020890	Gucy2e	1.048	0.592	-0.82	4.76E-03
ENSMUSG00000091264	Smim13	1.576	0.884	-0.83	3.72E-03
ENSMUSG00000036572	Upf3b	2.432	1.370	-0.83	3.90E-03
ENSMUSG00000020886	Dlg4	1.756	0.985	-0.83	3.90E-03
ENSMUSG00000043635	Adamts3	1.106	0.622	-0.83	4.48E-03
ENSMUSG00000022257	Laptm4b	2.154	1.202	-0.84	3.28E-03
ENSMUSG00000021983	Atp8a2	1.896	1.057	-0.84	3.38E-03
ENSMUSG00000033526	Ppip5k1	1.001	0.560	-0.84	3.85E-03
ENSMUSG00000029632	Ndufa4	6.281	3.474	-0.85	2.62E-03
ENSMUSG00000030096	Slc6a6	6.467	3.583	-0.85	2.68E-03
ENSMUSG00000039530	Tusc3	1.112	0.615	-0.85	2.99E-03
ENSMUSG00000050711	Scg2	1.189	0.661	-0.85	8.58E-03
ENSMUSG00000020547	Bzw2	1.427	0.786	-0.86	2.63E-03
ENSMUSG00000030064	Frmd4b	3.861	2.130	-0.86	2.65E-03
ENSMUSG00000022197	Pdzd2	2.735	1.496	-0.87	2.31E-03
ENSMUSG00000043987	Cep164	1.233	0.675	-0.87	2.51E-03
ENSMUSG00000026833	Olfm1	1.374	0.739	-0.89	1.70E-03
ENSMUSG00000031293	Rs1	3.469	1.873	-0.89	1.71E-03
ENSMUSG00000018427	Ypel2	1.206	0.645	-0.9	1.60E-03
ENSMUSG00000028524	Sgip1	6.738	3.580	-0.91	1.16E-03
ENSMUSG00000020458	Rtn4	4.248	2.265	-0.91	1.26E-03
ENSMUSG00000058254	Tspan7	1.697	0.901	-0.91	1.28E-03
ENSMUSG00000085438	1700020I14Rik	1.482	0.787	-0.91	1.30E-03
ENSMUSG00000041817	Fam169a	2.526	1.347	-0.91	1.34E-03
ENSMUSG00000051355	Commd1	1.316	0.701	-0.91	1.36E-03
ENSMUSG00000040490	Lrfn2	1.519	0.803	-0.92	1.33E-03
ENSMUSG00000025630	Hprt	1.361	0.713	-0.93	9.55E-04
ENSMUSG00000032292	Nr2e3	1.744	0.917	-0.93	1.13E-03
ENSMUSG00000048617	Rtbdn	5.807	3.038	-0.94	8.40E-04
ENSMUSG00000019978	Epb41l2	2.379	1.242	-0.94	8.77E-04

ENSMUSG00000036469	1-Mar	9.779	5.036	-0.96	6.00E-04
ENSMUSG00000058966	Fam57b	3.318	1.710	-0.96	6.26E-04
ENSMUSG00000021693	Kif2a	1.192	0.612	-0.96	6.38E-04
ENSMUSG00000024242	Map4k3	1.406	0.717	-0.97	5.77E-04
ENSMUSG00000050556	Kcnb1	3.922	1.990	-0.98	4.46E-04
ENSMUSG00000016319	Slc25a5	2.333	1.173	-0.99	3.72E-04
ENSMUSG00000013593	Ndufs2	1.856	0.928	-1	3.33E-04
ENSMUSG00000029048	Rer1	1.458	0.728	-1	3.36E-04
ENSMUSG00000059974	Ntm	6.364	3.167	-1.01	2.76E-04
ENSMUSG00000037697	Ddhd1	1.879	0.933	-1.01	2.97E-04
ENSMUSG00000053420	Gm4792	2.879	1.416	-1.02	2.17E-04
ENSMUSG00000041534	Rbp3	7.676	3.776	-1.02	2.18E-04
ENSMUSG00000060988	Galnt13	4.940	2.428	-1.03	2.22E-04
ENSMUSG00000024519	Cplx4	3.023	1.468	-1.04	1.62E-04
ENSMUSG00000001211	Agpat3	3.147	1.528	-1.04	1.64E-04
ENSMUSG00000035513	Ntng2	1.441	0.699	-1.04	1.94E-04
ENSMUSG00000056076	Eif3b	1.022	0.498	-1.04	2.23E-04
ENSMUSG00000032679	Cd59a	1.075	0.523	-1.04	2.25E-04
ENSMUSG00000002058	Unc119	15.363	7.435	-1.05	1.36E-04
ENSMUSG00000040554	Aipl1	6.684	3.238	-1.05	1.47E-04
ENSMUSG00000025026	Add3	1.086	0.526	-1.05	1.87E-04
ENSMUSG00000031558	Slit2	4.790	2.310	-1.05	7.86E-04
ENSMUSG00000031990	Jam3	1.285	0.617	-1.06	1.50E-04
ENSMUSG00000026239	Pde6d	1.947	0.930	-1.07	1.15E-04
ENSMUSG00000025860	Xiap	2.114	0.999	-1.08	9.26E-05
ENSMUSG00000044408	Sptssa	1.583	0.743	-1.09	7.94E-05
ENSMUSG00000023979	Guca1b	3.505	1.649	-1.09	8.30E-05
ENSMUSG00000059742	Kcnh7	1.045	0.492	-1.09	6.77E-04
ENSMUSG00000062209	Erbb4	2.263	1.049	-1.11	5.05E-04
ENSMUSG00000093904	Tomm20	4.644	2.140	-1.12	4.37E-05
ENSMUSG00000026004	Kansl1l	1.022	0.466	-1.13	4.81E-05
ENSMUSG00000079523	Tmsb10	7.550	3.396	-1.15	2.52E-05
ENSMUSG00000020598	Nrcam	1.928	0.868	-1.15	2.93E-05
ENSMUSG00000028125	Abca4	3.177	1.420	-1.16	2.15E-05
ENSMUSG00000032343	Impg1	1.938	0.865	-1.16	2.26E-05
ENSMUSG00000038718	Pbx3	2.308	1.034	-1.16	2.55E-05
ENSMUSG00000029330	Cds1	1.011	0.453	-1.16	2.91E-05
ENSMUSG00000040687	Madd	2.265	1.009	-1.17	1.98E-05
ENSMUSG00000061288	Taok3	1.110	0.492	-1.17	2.28E-05
ENSMUSG00000020089	Ppa1	1.693	0.748	-1.18	1.75E-05
ENSMUSG00000028013	Ppa2	1.425	0.631	-1.18	1.86E-05

ENSMUSG00000017344	Vtn	3.243	1.418	-1.19	1.23E-05
ENSMUSG00000042961	Egflam	1.590	0.695	-1.19	1.42E-05
ENSMUSG00000024992	Pde6c	5.654	2.441	-1.21	8.80E-06
ENSMUSG00000047547	Cltb	1.437	0.615	-1.22	7.73E-06
ENSMUSG00000020599	Rgs9	4.417	1.869	-1.24	4.91E-06
ENSMUSG00000027589	Pcmtd2	1.948	0.822	-1.24	5.41E-06
ENSMUSG00000057132	Rpgrip1	7.836	3.291	-1.25	3.87E-06
ENSMUSG00000079550	Mpp4	2.244	0.943	-1.25	4.44E-06
ENSMUSG00000029276	Glmn	2.047	0.861	-1.25	4.71E-06
ENSMUSG00000023982	Guca1a	15.807	6.620	-1.26	3.54E-06
ENSMUSG00000040111	Gramd1b	1.858	0.773	-1.26	3.72E-06
ENSMUSG00000023978	Prph2	11.470	4.754	-1.27	2.62E-06
ENSMUSG00000003500	Impdh1	2.418	1.005	-1.27	3.18E-06
ENSMUSG00000024842	Cabp4	1.699	0.705	-1.27	3.36E-06
ENSMUSG00000027350	Chgb	2.376	0.979	-1.28	2.65E-06
ENSMUSG00000113262	Gm48551	1.604	0.660	-1.28	2.77E-06
ENSMUSG00000024211	Grm8	1.530	0.626	-1.29	3.17E-06
ENSMUSG00000032181	Scg3	2.084	0.838	-1.31	1.33E-06
ENSMUSG00000034837	Gnat1	6.716	2.704	-1.31	1.41E-06
ENSMUSG00000112404	AC159282.1	1.855	0.747	-1.31	1.60E-06
ENSMUSG00000023439	Gnb3	12.775	5.121	-1.32	1.05E-06
ENSMUSG00000071654	Uqcc3	1.245	0.499	-1.32	1.35E-06
ENSMUSG00000020444	Guk1	5.809	2.310	-1.33	8.42E-07
ENSMUSG00000039375	Wdr17	2.434	0.959	-1.34	7.73E-07
ENSMUSG00000002459	Rgs20	6.487	2.538	-1.35	5.68E-07
ENSMUSG00000021745	Ptprg	1.889	0.741	-1.35	7.74E-07
ENSMUSG00000056043	Rgs9bp	1.149	0.451	-1.35	9.12E-07
ENSMUSG00000029663	Gngt1	23.055	8.977	-1.36	4.34E-07
ENSMUSG00000063626	Unc5d	1.399	0.546	-1.36	1.84E-06
ENSMUSG00000052613	Pcdh15	30.155	11.561	-1.38	2.89E-07
ENSMUSG00000092349	Platr17	4.105	1.573	-1.38	2.93E-07
ENSMUSG00000026123	Plekhb2	1.366	0.525	-1.38	4.37E-07
ENSMUSG00000020907	Rcvrn	12.573	4.814	-1.39	2.78E-07
ENSMUSG00000032087	Dscaml1	3.205	1.203	-1.41	1.89E-07
ENSMUSG00000030657	Xylt1	1.121	0.423	-1.41	2.94E-07
ENSMUSG00000091537	Tma7	20.024	7.360	-1.44	7.59E-08
ENSMUSG00000035504	Reep6	1.056	0.386	-1.45	2.06E-07
ENSMUSG00000028461	Ccdc107	2.133	0.773	-1.46	6.23E-08
ENSMUSG00000037446	Tulp1	8.729	3.125	-1.48	3.40E-08
ENSMUSG00000079055	Slc8a3	2.341	0.815	-1.52	1.99E-08
ENSMUSG00000091908	Gm17231	1.226	0.428	-1.52	2.61E-08

ENSMUSG00000046049	Rp1l1	1.365	0.472	-1.53	1.84E-08
ENSMUSG00000032258	Lca5	1.160	0.402	-1.53	3.08E-08
ENSMUSG00000071648	Rom1	10.766	3.671	-1.55	7.73E-09
ENSMUSG00000042363	Lgalsl	2.589	0.878	-1.56	7.59E-09
ENSMUSG00000021803	Cdhr1	2.294	0.780	-1.56	8.95E-09
ENSMUSG00000005583	Mef2c	1.632	0.545	-1.58	6.57E-09
ENSMUSG00000006007	Pdc	13.694	4.446	-1.62	1.52E-09
ENSMUSG00000000628	Hk2	4.086	1.286	-1.67	6.05E-10
ENSMUSG00000024227	Pdzph1	3.264	0.991	-1.72	2.26E-10
ENSMUSG00000049811	Fam161a	2.056	0.599	-1.78	5.08E-11
ENSMUSG00000033701	Acbd6	3.060	0.868	-1.82	1.67E-11
ENSMUSG00000029672	Fam3c	1.038	0.285	-1.86	2.30E-11
ENSMUSG00000029064	Gnb1	6.584	1.749	-1.91	1.69E-12
ENSMUSG00000030324	Rho	29.917	7.887	-1.92	1.39E-12
ENSMUSG00000075410	Prcd	4.030	1.045	-1.95	6.24E-13
ENSMUSG00000034813	Grip1	1.217	0.314	-1.95	4.32E-12
ENSMUSG00000056494	Cngb3	11.012	2.806	-1.97	2.99E-13
ENSMUSG00000021221	Dpf3	1.660	0.424	-1.97	7.00E-13
ENSMUSG00000040632	Nrl	2.422	0.613	-1.98	4.63E-13
ENSMUSG00000024856	Cdk2ap2	1.752	0.427	-2.03	8.61E-14
ENSMUSG00000038811	Gngt2	20.008	4.627	-2.11	7.38E-15
ENSMUSG00000032064	Dixdc1	2.508	0.574	-2.13	5.92E-15
ENSMUSG00000084826	AI847159	2.192	0.478	-2.2	9.50E-16
ENSMUSG00000025386	Pde6g	12.989	2.804	-2.21	4.37E-16
ENSMUSG00000030956	Fam53b	1.026	0.220	-2.22	3.38E-15
ENSMUSG00000040420	Cdh18	15.432	3.222	-2.26	1.26E-16
ENSMUSG00000025340	Rabgef1	1.178	0.242	-2.28	6.05E-16
ENSMUSG00000054206	Gzmm	1.909	0.389	-2.29	1.96E-16
ENSMUSG00000006289	Osgep	2.467	0.499	-2.31	4.47E-17
ENSMUSG00000047034	Ankrd33	1.144	0.230	-2.31	2.04E-16
ENSMUSG00000039672	Kcne2	2.399	0.453	-2.4	4.37E-18
ENSMUSG00000041992	Rapgef5	1.758	0.332	-2.4	1.03E-17
ENSMUSG00000079834	Tmlhe	1.440	0.254	-2.5	8.91E-19
ENSMUSG00000029769	Ccdc136	3.336	0.542	-2.62	7.33E-21
ENSMUSG00000074365	Crxos	1.976	0.312	-2.66	5.12E-21
ENSMUSG00000047907	Tshz2	1.553	0.245	-2.66	1.74E-20
ENSMUSG00000024558	Mapk4	1.143	0.170	-2.74	3.19E-21
ENSMUSG00000000308	Ckmt1	1.501	0.202	-2.89	8.98E-24
ENSMUSG00000022836	Mylk	2.947	0.387	-2.93	1.15E-24
ENSMUSG00000028391	Wdr31	1.045	0.137	-2.93	8.57E-24
ENSMUSG00000027384	Ndufaf5	1.361	0.175	-2.95	1.95E-24

ENSMUSG00000009108	Gnat2	10.555	1.352	-2.96	5.65E-26
ENSMUSG00000021396	Nxnl2	1.547	0.183	-3.08	3.08E-26
ENSMUSG00000064330	Pde6h	33.988	3.824	-3.15	2.00E-28
ENSMUSG00000035283	Adrb1	1.228	0.129	-3.24	5.36E-28
ENSMUSG00000060890	Arr3	23.535	2.191	-3.43	1.47E-32
ENSMUSG00000058831	Opn1sw	29.180	2.655	-3.46	2.61E-32
ENSMUSG00000031394	Opn1mw	16.245	1.305	-3.64	2.15E-35

Supplementary Table S3. DEGs in phototransduction pathway in cones after T3 treatment.

Symbol	log2FC	Expr p-value
ARR3	-3.425	1.47E-32
CNGB3	-1.973	2.99E-13
GNAT1	-1.313	1.41E-06
GNAT2	-2.965	5.65E-26
GNB1	-1.913	1.69E-12
GNB3	-1.319	1.05E-06
GNB5	-0.71	1.68E-02
GNGT1	-1.361	4.34E-07
GNGT2	-2.112	7.38E-15
GUCA1A	-1.256	3.54E-06
GUCA1B	-1.088	8.30E-05
GUCY1A1	1.337	4.45E-08
GUCY2D	-0.822	4.76E-03
OPN1MW	-3.637	2.15E-35
OPN1SW	-3.459	2.61E-32
PDC	-1.623	1.52E-09
PDE6B	-0.686	2.30E-02
PDE6C	-1.212	8.80E-06
PDE6D	-1.066	1.15E-04
PDE6G	-2.212	4.37E-16
PDE6H	-3.152	2.00E-28
RCVRN	-1.385	2.78E-07
RGS9	-1.24	4.91E-06
RGS9BP	-1.348	9.12E-07
RHO	-1.924	1.39E-12

Supplementary Table S4. DEGs in oxidative phosphorylation in cones after T3 treatment.

Symbol	log2FC	Expr p-value
ATP5MC1	-0.618	4.54E-02
COX8A	-0.733	1.24E-02
MT-ATP6	-0.668	2.64E-02
MT-CO1	-0.79	6.12E-03
MT-CO2	-0.625	4.15E-02
MT-CO3	-0.784	6.67E-03
MT-CYB	-0.813	4.59E-03
MT-ND4	-0.7	1.83E-02
MT-ND5	-0.718	1.51E-02
NDUFA4	-0.855	2.62E-03
NDUFA10	-0.708	1.79E-02
NDUFA12	-0.648	3.41E-02
NDUFS2	-1	3.33E-04

Supplementary Table S5. DEGs in mitochondrial dysfunction in cones after T3 treatment.

Symbol	log2FC	Expr p-value
APP	1.369	1.36E-08
ATP5MC1	-0.618	4.54E-02
CACNA1D	1.484	7.40E-10
CACNA1F	-0.758	1.01E-02
CACNA1H	1.151	3.88E-06
CACNA2D1	2.239	6.65E-22
COX8A	-0.733	1.24E-02
MT-ATP6	-0.668	2.64E-02
MT-CO1	-0.79	6.12E-03
MT-CO2	-0.625	4.15E-02
MT-CO3	-0.784	6.67E-03
MT-CYB	-0.813	4.59E-03
MT-ND4	-0.7	1.83E-02
MT-ND5	-0.718	1.51E-02
NDUFA4	-0.855	2.62E-03
NDUFA10	-0.708	1.79E-02
NDUFA12	-0.648	3.41E-02
NDUFS2	-1	3.33E-04
PRKN	0.642	2.30E-02
RHOT1	-0.648	3.39E-02
TOMM20	-1.118	4.37E-05

Supplementary Table S6. DEGs in EIF2 Signaling in cones after T3 treatment.

Symbol	log2FC	Expr p-value
AKT3	-0.664	2.93E-02
ATF3	4.07	1.68E-57
DDIT3	1.541	1.52E-10
EIF5	0.901	4.63E-04
EIF2S2	0.72	8.27E-03
EIF3B	-1.036	2.23E-04
EIF3C	0.619	3.04E-02
RPL10	0.969	1.33E-04
RPL12	0.681	1.34E-02
RPL13	0.789	2.64E-03
RPL23	0.629	2.46E-02
RPL26	0.699	1.01E-02
RPL37	0.736	5.91E-03
RPL38	0.676	1.36E-02
RPL18A	0.572	4.90E-02
Rpl34	0.702	9.71E-03
RPL35A	0.702	9.63E-03
RPS12	0.665	1.66E-02
RPS16	0.581	4.39E-02
RPS19	0.672	1.50E-02
RPS23	0.686	1.21E-02
RPS24	0.602	3.43E-02
RPS26	0.71	9.08E-03
RPS28	0.712	8.45E-03
RPS27A	0.584	4.23E-02
XIAP	-1.081	9.26E-05

Supplementary Table S7. DEGs in CREB signaling in cones after T3 treatment.

Symbol	log2FC	Expr p-value
ADCY2	1.025	5.46E-05
ADGRB3	0.968	1.25E-04
ADGRL3	2.124	1.98E-19
ADRB1	-3.24	5.36E-28
AKT3	-0.664	2.93E-02
CACNA1D	1.484	7.40E-10
CACNA1F	-0.758	1.01E-02
CACNA1H	1.151	3.88E-06
CACNA2D1	2.239	6.65E-22
CAMK4	1.79	5.58E-14
CHRM3	1.959	6.57E-17
GNAT1	-1.313	1.41E-06
GNAT2	-2.965	5.65E-26
GNB1	-1.913	1.69E-12
GNB3	-1.319	1.05E-06
GNB5	-0.71	1.68E-02
GNG13	0.831	1.86E-03
GRIA1	1.846	1.32E-14
GRIA2	0.785	3.72E-03
GRID1	1.584	4.90E-11
GRIK1	1.882	8.96E-16
GRM7	1.256	4.35E-07
GRM8	-1.289	3.17E-06
GUCY1A1	1.337	4.45E-08
LGR5	0.972	1.63E-04
OPN1LW	-3.637	2.15E-35
OPN1SW	-3.459	2.61E-32
PLCB4	1.123	4.48E-06
PLCL1	1.127	5.28E-06
PRKCA	0.778	4.00E-03
PRKCB	1.557	9.24E-11
RHO	-1.924	1.39E-12

Supplementary Table S8. DEGs in rods after T3 treatment.

Feature ID	Gene name	Average expression level in control	Average expression level in T3 treatment	Log2 FC	P-Value
ENSMUSG00000086503	Xist	0.000	1.043	12.44	4.45E-245
ENSMUSG00000006204	5430419D17Rik	0.105	1.453	3.79	1.17E-53
ENSMUSG00000023439	Gnb3	0.194	1.752	3.17	1.30E-39
ENSMUSG00000075256	Cerkl	0.185	1.332	2.85	9.40E-33
ENSMUSG00000021557	Agtpbp1	0.274	1.201	2.13	2.93E-19
ENSMUSG00000025496	Drd4	0.435	1.253	1.53	2.65E-10
ENSMUSG00000034701	Neurod1	0.586	1.454	1.31	8.89E-08
ENSMUSG00000022708	Zbtb20	0.642	1.482	1.21	1.05E-06
ENSMUSG00000040003	Magi2	1.375	3.095	1.17	2.42E-06
ENSMUSG00000021684	Pde8b	0.503	1.088	1.11	8.30E-06
ENSMUSG00000004535	Tax1bp1	0.509	1.074	1.08	1.74E-05
ENSMUSG00000039145	Camk1d	1.148	2.220	0.95	2.06E-04
ENSMUSG00000009470	Tnpo1	0.656	1.264	0.95	2.26E-04
ENSMUSG00000021779	Thrb	0.717	1.295	0.85	1.13E-03
ENSMUSG00000060743	H3f3a	1.311	2.214	0.76	5.09E-03
ENSMUSG00000048617	Rtbdn	0.961	1.520	0.66	1.84E-02
ENSMUSG00000064115	Cadm2	6.430	9.637	0.58	4.53E-02
ENSMUSG00000085794	Vax2os	1.342	0.867	-0.63	3.15E-02
ENSMUSG00000024575	Pde6a	2.258	1.440	-0.65	2.56E-02
ENSMUSG00000028524	Sgip1	1.691	1.062	-0.67	1.96E-02
ENSMUSG00000050556	Kcnb1	1.572	0.980	-0.68	1.71E-02
ENSMUSG00000031789	Cngb1	1.287	0.796	-0.69	1.48E-02
ENSMUSG00000029632	Ndufa4	1.250	0.767	-0.70	1.29E-02
ENSMUSG00000028125	Abca4	1.127	0.666	-0.76	6.28E-03
ENSMUSG00000032087	Dscaml1	1.125	0.655	-0.78	4.73E-03
ENSMUSG00000039375	Wdr17	1.009	0.554	-0.86	1.38E-03
ENSMUSG00000023978	Prph2	5.191	2.834	-0.87	1.21E-03
ENSMUSG00000042757	Tmem108	1.295	0.698	-0.89	9.04E-04
ENSMUSG00000001211	Agpat3	1.433	0.763	-0.91	6.71E-04
ENSMUSG00000091537	Tma7	4.375	2.324	-0.91	6.47E-04
ENSMUSG00000029491	Pde6b	1.871	0.976	-0.94	4.14E-04
ENSMUSG00000068205	Macrod2	1.082	0.553	-0.97	2.51E-04
ENSMUSG00000000628	Hk2	1.282	0.644	-0.99	1.65E-04
ENSMUSG00000030096	Slc6a6	1.401	0.669	-1.07	4.44E-05
ENSMUSG00000057132	Rpgrip1	2.198	1.035	-1.09	2.99E-05
ENSMUSG00000029663	Gngt1	8.574	4.001	-1.10	2.34E-05

ENSMUSG00000106379	Lhfpl3	1.024	0.475	-1.11	2.00E-05
ENSMUSG00000032292	Nr2e3	1.328	0.608	-1.13	1.40E-05
ENSMUSG00000002058	Unc119	4.286	1.911	-1.16	6.48E-06
ENSMUSG00000023979	Guca1b	2.429	1.046	-1.22	2.33E-06
ENSMUSG00000006007	Pdc	6.549	2.805	-1.22	1.97E-06
ENSMUSG00000034837	Gnat1	4.296	1.825	-1.23	1.54E-06
ENSMUSG00000019978	Epb41l2	1.451	0.613	-1.24	1.28E-06
ENSMUSG00000027589	Pcmtd2	1.077	0.429	-1.33	2.06E-07
ENSMUSG00000071648	Rom1	4.615	1.727	-1.42	2.47E-08
ENSMUSG00000075410	Prcd	1.133	0.408	-1.47	6.69E-09
ENSMUSG00000025386	Pde6g	4.168	1.394	-1.58	4.14E-10
ENSMUSG00000024227	Pdzph1	1.845	0.608	-1.60	2.40E-10
ENSMUSG00000030324	Rho	18.087	5.169	-1.81	7.20E-13
ENSMUSG00000029064	Gnb1	4.820	1.086	-2.15	1.62E-17
ENSMUSG00000040632	Nrl	1.763	0.389	-2.18	6.19E-18

Supplementary Table S9. Comparison of proportion of major retinal cell types identified by different studies.

Cell type	Percentage of retinal cells (Jeon et al. 1998)	Percentage of retinal cells (Macosko et al. 2015)	Percentage of retinal cells (present study)
rods	79.9	65.6	76
cones	2.1	4.2	4.1
ganglion cells	0.5	1	0.6
bipolar cells	7.3	14	9.3
amacrine cells	7	9.9	1.4
muller	2.8	3.6	5
horizontal cells	0.5	0.6	0.6
microglial		0.2	0.5
Astrocyte		0.1	1.9

Supplementary Table S10. Shared DEGs in rods and cones after T3 treatment.

Gene	log2FC in cones	log2FC in rods	Expr p-value in cones	Expr p-value in rods	Direction
NRL	-1.98	-2.18	4.63E-13	6.19E-18	same
GNB1	-1.91	-2.15	1.69E-12	1.62E-17	same
RHO	-1.92	-1.81	1.39E-12	7.20E-13	same
Pdzph1	-1.72	-1.60	2.26E-10	2.40E-10	same
PDE6G	-2.21	-1.58	4.37E-16	4.14E-10	same
Prcd	-1.95	-1.47	6.24E-13	6.69E-09	same
ROM1	-1.55	-1.42	7.73E-09	2.47E-08	same
PCMTD2	-1.25	-1.33	5.41E-06	2.06E-07	same
EPB41L2	-0.94	-1.24	8.77E-04	1.28E-06	same
GNAT1	-1.31	-1.24	1.41E-06	1.54E-06	same
PDC	-1.62	-1.22	1.52E-09	1.97E-06	same
GUCA1B	-1.09	-1.22	8.30E-05	2.33E-06	same
UNC119	-1.05	-1.17	1.36E-04	6.48E-06	same
NR2E3	-0.93	-1.13	1.13E-03	1.40E-05	same
GNGT1	-1.36	-1.10	4.34E-07	2.34E-05	same
RPGRIP1	-1.25	-1.09	3.87E-06	2.99E-05	same
SLC6A6	-0.85	-1.07	2.68E-03	4.44E-05	same
HK2	-1.67	-0.99	6.05E-10	1.65E-04	same
PDE6B	-0.69	-0.94	2.30E-02	4.14E-04	same
TMA7	-1.44	-0.91	7.59E-08	6.47E-04	same
AGPAT3	-1.04	-0.91	1.64E-04	6.71E-04	same
PRPH2	-1.27	-0.87	2.62E-06	1.21E-03	same
WDR17	-1.34	-0.87	7.73E-07	1.38E-03	same
DSCAML1	-1.41	-0.78	1.89E-07	4.73E-03	same
ABCA4	-1.16	-0.76	2.15E-05	6.28E-03	same
NDUFA4	-0.86	-0.71	2.62E-03	1.29E-02	same
KCNB1	-0.98	-0.68	4.46E-04	1.71E-02	same
SGIP1	-0.91	-0.67	1.16E-03	1.96E-02	same
RTBDN	-0.94	0.66	8.40E-04	1.84E-02	opposite
TNPO1	1.00	0.95	7.09E-05	2.26E-04	same
ZBTB20	1.01	1.21	5.10E-05	1.05E-06	same
AGTPBP1	1.10	2.13	8.16E-06	2.93E-19	same
CERKL	1.47	2.85	7.97E-10	9.40E-33	same
GNB3	-1.32	3.17	1.05E-06	1.30E-39	opposite
Cdcp3	1.76	3.79	6.92E-14	1.17E-53	same
XIST	15.46	12.44	1.39E-274	4.45E-245	same

Supplementary Table S11. PCR primers used.

Gene	Forward	Reverse
Arr3	ATGAACAAGGAGCTTCTGGG	CAGGAGATGGCTTTGGATGG
Cdkn1a	CTTGCACTCTGGTGTCTGAG	GCACTTCAGGGTTTTCTCTTG
Gnb1	TCTGGGAATTATGTGGCCTG	TGATTGTCATCCAGGAACCG
Gngt2	CCAAGCAAAGGAAGCCAAG	CATCCTCAACAGCTCCTTCTC
Hk2	TTTCACCTTCTCCTTCCCTTG	CACATCTTCACCCTCGCAG
Hprt1	GCAAACTTTGCTTTCCCTGGTT	CAAGGGCATATCCAACAACA
Opn1mw	GCTACTTCGTTCTGGGACAC	CAAATCTCACATTGCCAAAGGG
OPN1sw	TGAAAGAGTGGGAAAGGATGG	CACCAAGACAGAAAGAGTAGGG
Pde6a	AGTACGAAGCCAAGATGAAGG	AACAGGACTTAGATGCAGGTG
Pde6b	TGGAGCTAGTCAAATGTGGC	GAGTAGGGTAAACATGGTCTGG
Pde6g	GCAAACAAGGCAGTTCAAGAG	GGGCAGATGACGGTGATATC
Rho	GGGAGAATCACGCTATCATGG	GTCAATCCCGCATGAACATTG
Xist	TAAGGACTACTTAACGGGCT	TACTCAGACATTCCCTGGCA
